# Spatial compartmentalization of melanoma cell states reveals CAF-associated tumor cells with enhanced proliferative capacity

**DOI:** 10.1371/journal.pone.0358235

**Published:** 2026-09-15

**Authors:** Weilong Zheng, Huanhuan Zheng

**Affiliations:** 1 Department of Plastic and Cosmetic Surgery, Jinjiang Municipal Hospital (Shanghai Sixth People’s Hospital Fujian), Jinjiang, China; 2 Department of Clinical Nutrition, Ji’an Central People’s Hospital, Ji’an, China; Sichuan University, CHINA

## Abstract

**Background:**

Cancer-associated fibroblasts (CAFs) are key components of the melanoma tumor microenvironment and have been implicated in immune evasion and therapy resistance. However, the spatial relationships between CAFs, extracellular matrix (ECM) remodeling, and immune exclusion remain incompletely characterized.

**Methods:**

We integrated single-cell RNA sequencing (scRNA-seq) and spatial transcriptomics from primary melanoma samples (892 regions of interest) to investigate the landscape of CAF-immune interactions. CAF scores were calculated based on five fibroblast-associated genes. Differential expression analysis, Spearman correlation, and cell state subclustering were performed to characterize the spatial organization of the tumor microenvironment. Findings were examined using TCGA bulk RNA-seq data and drug sensitivity was assessed using the GDSC database.

**Results:**

CAF-high regions exhibited significantly lower immune scores compared to CAF-low regions (ρ = −0.442, p < 0.001), with enrichment of ECM-related genes (COL1A1, COL1A2, DCN, FBLN1) and downregulation of immune genes (PTPRC, CD4, CD8A). Spatial co-expression of FN1 and its integrin receptor subunit ITGB1 was observed (ρ = 0.100, p = 0.003), supporting a potential role for ECM-receptor signaling in immune exclusion. Six distinct melanoma cell states were identified, with CAF-associated tumor states (State 4) exhibiting significantly higher proliferative capacity than immune-associated states (State 5) (p = 0.007). The CAF signature correlated with cisplatin sensitivity (ρ = −0.627, p < 0.001) and trametinib resistance (ρ = 0.354, p = 0.009).

**Conclusions:**

This study provides a spatial atlas of CAF-immune interactions in melanoma. CAF abundance is negatively correlated with immune infiltration at the spatial level, with ECM remodeling and FN1-ITGB1 signaling potentially mediating this immune exclusion. The CAF signature showed correlations with drug sensitivity in cell line models, warranting further investigation in CAF-containing systems.

## 1 Introduction

Cutaneous melanoma, the most aggressive form of skin cancer, accounts for approximately 80% of skin cancer-related deaths despite representing only 4–5% of all skin cancer cases [1,2]. Over the past five decades, the global incidence of melanoma has steadily increased, with an estimated 104,960 new cases and 8,430 deaths projected in the United States for 2025 alone [1,2]. While early-stage localized melanoma is often curable by surgical resection, the prognosis for patients with metastatic disease remains poor [2,3]. This stark disparity underscores the urgent need for improved therapeutic strategies and a deeper understanding of the biological mechanisms driving melanoma progression.

The advent of immune checkpoint inhibitors (ICIs) targeting CTLA-4 and PD-1/PD-L1 has revolutionized the treatment of advanced melanoma, achieving durable responses in a substantial subset of patients [2,4]. However, primary or acquired resistance to ICIs occurs in approximately 40–60% of patients, limiting the overall clinical benefit [2,5,6]. Resistance mechanisms are multifactorial, encompassing tumor-intrinsic factors as well as tumor-extrinsic factors related to the composition and functional state of the tumor microenvironment [3,4,6]. Among these, the physical exclusion of cytotoxic T cells from the tumor core—termed “immune exclusion”—has emerged as a critical barrier to effective immunotherapy [5–7].

Cancer-associated fibroblasts (CAFs) are a major cellular component of the melanoma tumor microenvironment and have been increasingly recognized as key orchestrators of immune exclusion [5,8,9]. Activated CAFs deposit dense extracellular matrix (ECM) components, including collagens and fibronectin, which form a physical barrier that impedes T cell infiltration [5,6,10,11]. Beyond their structural role, CAFs secrete immunosuppressive cytokines and chemokines, such as TGF-β, IL-6, and CXCL12, which promote the recruitment of regulatory T cells and myeloid-derived suppressor cells while directly suppressing effector T cell function [7,12–14]. Recent single-cell and spatial transcriptomic studies have revealed remarkable heterogeneity within CAF populations, with specific CAF subtypes—such as matrix CAFs (mCAFs) and immunomodulatory CAFs (iCAFs)—playing distinct roles in ECM remodeling and immune modulation [5,9,15,16]. Moreover, CAF-derived factors have been implicated in resistance to targeted therapies and ICIs, highlighting the clinical relevance of targeting the stromal compartment [12,14,17].

The application of single-cell RNA sequencing (scRNA-seq) and spatial transcriptomics has transformed our ability to dissect cellular heterogeneity and spatial organization within the tumor microenvironment [15,16,18]. scRNA-seq enables high-resolution characterization of cell states and lineages, while spatial transcriptomics preserves the spatial context of gene expression, allowing for the direct visualization of cell-cell interactions within the tissue architecture [9,11,18]. Integrating these two approaches has proven particularly powerful for studying CAF heterogeneity and their spatial relationships with immune cells in various cancers, including melanoma [5,9,15,16]. However, the precise spatial relationships between CAF abundance, ECM remodeling, and immune exclusion in the melanoma microenvironment remain incompletely characterized.

In this study, we integrated scRNA-seq and spatial transcriptomics data to investigate the role of CAFs in the melanoma tumor microenvironment. Our objectives were to characterize the spatial relationship between CAF abundance and immune infiltration, identify ECM-related genes and ligand-receptor pairs potentially mediating CAF-driven immune exclusion, examine the spatial compartmentalization of melanoma cell states in relation to CAF-rich versus immune-rich niches, and evaluate the clinical relevance and drug sensitivity associations of the CAF signature using independent bulk RNA-seq data.

## 2 Methods

### 2.1 Study design and data sources

This was a retrospective cohort study. This study integrated scRNA-seq and spatial transcriptomics to investigate the role of CAFs in the melanoma tumor microenvironment, with subsequent validation using bulk RNA-seq data from The Cancer Genome Atlas (TCGA). The scRNA-seq and spatial transcriptomics data were obtained from Gene Expression Omnibus (GEO) under accession numbers GSE291160 and GSE291159, respectively [19]. TCGA skin cutaneous melanoma (SKCM) data were downloaded from the GDC data portal, including RNA-seq expression (STAR-TPM normalized) and corresponding clinical information.

### 2.2 Study setting, participants, and eligibility criteria

Study setting: The original scRNA-seq and spatial transcriptomics data were collected as part of a study approved by the New York University Institutional Review Board (IRB no. s16–00122), with informed consent obtained from all participants [19]. Spatial transcriptomics was performed on de-identified archival tissue samples, for which ethical approval was waived. Samples were collected at multiple academic medical centers in the United States, including New York University, University of California San Diego, and Memorial Sloan Kettering Cancer Center. The exact dates of sample collection were not specified in the original publication; however, the study was published in 2025. TCGA-SKCM data involved multi-center sample collection across North America between approximately 2000 and 2010. The scRNA-seq dataset comprised three samples based on data availability in the original study [19].

Eligibility criteria for spatial transcriptomics data: GeoMx digital spatial profiling was performed on formalin-fixed paraffin-embedded sections from eight primary melanoma samples [19]. Regions of interest (ROIs) were selected based on immunofluorescence markers for keratinocytes (PanCK), melanoma cells (S100B, PMEL), and immune cells (CD45), ensuring representation of pure or mixed cell type areas. A total of 892 ROIs were included. ROIs with insufficient RNA quality or those failing quality control metrics were excluded by the original authors.

Eligibility criteria for TCGA validation cohort: TCGA-SKCM data included all available samples with complete RNA-seq expression profiles and corresponding clinical information. Samples with missing survival data or key clinical covariates were excluded, resulting in 316 samples for analysis. Of the original 472 TCGA-SKCM samples, 156 were excluded due to missing survival data (n = 98) or incomplete clinical covariates (n = 58).

This study only utilized publicly available anonymized datasets. Original studies involving human participants were reviewed and approved by their respective Institutional Review Boards. As this work involved the re-analysis of existing de-identified data, it did not constitute human subject research requiring additional ethical approval.

### 2.3 Single-cell RNA-seq data processing

Raw scRNA-seq data from three primary melanoma samples were processed using the inDrop platform. Quality control was performed to retain cells with ≥500 unique molecular identifiers (UMIs), ≥ 200 detected genes, and <20% mitochondrial reads. After filtering, 1,832 high-quality cells were retained for downstream analysis. Data normalization was performed using the LogNormalize method with a scale factor of 10,000 [20]. Principal component analysis (PCA) was conducted on the top 2,000 highly variable genes, and uniform manifold approximation and projection (UMAP) was used for dimensional reduction with the first 20 principal components [21]. The number of principal components (PCs) was selected based on the elbow plot of variance explained. Clustering resolution was set to 0.5 to separate major cell types. UMAP visualization was performed with default parameters (n.neighbors = 30, min.dist = 0.3). Batch effects were corrected using the Harmony algorithm prior to downstream analysis.

### 2.4 Cell type annotation

Cell types were identified using a module score approach based on canonical marker genes. Four major cell types were annotated: melanocytes (PMEL, TYR, MLANA, MITF, SOX10), keratinocytes (KRT5, KRT14, KRT17, DSC3, DSP), immune cells (PTPRC, CD68, CD4, CD8A, CSF1R), and fibroblasts (COL1A2, DCN, COL6A2, COL1A1, FBLN1). Cells with module scores below a threshold of 0.1 for all cell types were classified as “Unknown.” Module scores were calculated using the Seurat AddModuleScore function, which computes the average expression of each gene set minus the average expression of a control set. The threshold of 0.1 was chosen to minimize false-positive cell type assignments.

### 2.5 Spatial transcriptomics processing

GeoMx digital spatial profiling data from eight primary melanoma samples generated transcriptomic profiles for 892 regions of interest (ROIs) [22]. Raw count data were normalized using log2 transformation with a pseudo-count of 1. Cell type-specific module scores were calculated for each ROI using the same marker gene sets as in scRNA-seq. Unsupervised clustering was performed using the Louvain algorithm with a resolution parameter of 0.5, identifying 10 distinct spatial clusters. This resolution was chosen to yield biologically interpretable spatial clusters. Louvain clustering was performed using default parameters. Dimensionality reduction for spatial data was based on the first 10 PCs, which explained the majority of variance.

### 2.6 CAF score calculation

The CAF score was defined as the average expression of five fibroblast-associated genes: COL1A2, DCN, COL6A2, COL1A1, and FBLN1. These genes were selected because they were among the most highly expressed and consistently detected fibroblast markers across all three scRNA-seq samples, and they represent core ECM-related genes commonly associated with CAF activation. While we acknowledge that this score captures a pan-CAF signature, we also validated the CAF grouping using alternative CAF markers (FAP, PDPN, THY1, POSTN), confirming the robustness of our stratification. For spatial data, CAF scores were calculated at the ROI level. For TCGA data, CAF scores were computed for each sample using the same gene set. Samples were stratified into high-CAF and low-CAF groups based on the median CAF score.

### 2.7 Definition of outcome variables

Immune score: The immune score for each ROI was calculated as the average expression of immune cell marker genes (PTPRC, CD68, CD4, CD8A, CSF1R), selected based on their established roles as immune lineage markers, using the same module score approach described for cell type annotation (section 2.4).

Proliferation score: The proliferation score for melanoma cells was calculated as the average expression of cell proliferation-associated genes, including MKI67, MCM4, and PCNA, selected based on their known roles in cell cycle progression, using the Seurat AddModuleScore function.

Drug sensitivity: Drug sensitivity was assessed using log-transformed IC50 values obtained from the Genomics of Drug Sensitivity in Cancer (GDSC) database [23]. Lower IC50 values indicate higher sensitivity; higher IC50 values indicate resistance.

### 2.8 Desmosome score calculation

Desmosome score was calculated as the average expression of 12 desmosome-related genes: DSC1, DSC2, DSC3, DSG1, DSG2, DSG3, DSP, PKP1, PKP2, PKP3, JUP, and PERP. This gene set was selected based on established desmosome gene annotations. For each cell, the desmosome score was computed using log-normalized expression values. Comparisons of desmosome scores across cell types were performed using Wilcoxon rank-sum test.

### 2.9 Differential gene expression analysis

Differential gene expression between high-CAF and low-CAF groups was performed using the Wilcoxon rank-sum test implemented in the Seurat FindMarkers function [20]. Genes with |log2 fold change| > 0.5 and adjusted p-value < 0.05 were considered statistically significant. Volcano plots were generated using ggplot2 with genes of interest labeled. The five genes used for CAF score calculation were not excluded from differential expression analysis, as the goal was to characterize the full transcriptional landscape of CAF-high versus CAF-low regions.

### 2.10 Melanoma cell subclustering

Melanoma cells were extracted from the scRNA-seq dataset and re-clustered using the Louvain algorithm (resolution = 0.3), resulting in six distinct subclusters. Marker genes for each subcluster were identified using FindAllMarkers with a log2 fold change threshold of 0.5 and minimum percentage of 0.25. A lower resolution (0.3) was chosen to avoid over-splitting the relatively small number of melanoma cells (n = 588). The resolution parameter was selected by testing values from 0.1 to 0.8, and resolution = 0.3 was chosen because it yielded clusters with distinct and biologically interpretable marker gene expression profiles, without over-splitting. UMAP was performed with n.neighbors = 20 and min.dist = 0.3 to better capture the structure of this subset. Dimensionality reduction was based on the first 10 PCs.

### 2.11 Statistical analysis

All statistical analyses were performed using R version 4.5.2. Spearman correlation was used for non-parametric correlation analyses. Wilcoxon rank-sum test was applied for two-group comparisons, and Kruskal-Wallis test for multi-group comparisons. Survival analysis was performed using the Kaplan-Meier method with log-rank test. All tests were two-sided, and p-values < 0.05 were considered statistically significant. Multiple testing corrections were applied using the Benjamini-Hochberg method where indicated.

Potential confounders were not formally adjusted for in the primary analyses due to the exploratory nature of the study; however, we assessed the association between CAF score and tumor stage in the TCGA cohort and found no significant correlation (Kruskal-Wallis test, p = 0.395), suggesting that stage is unlikely to be a major confounder. Effect modification was not assessed. Subgroup analyses were performed by tumor stage and by cell type (keratinocyte vs melanocyte ROIs)

There were no missing data for the key variables (CAF score, immune score, proliferation score) in the spatial transcriptomics dataset. For the TCGA dataset, samples with missing survival data or key clinical covariates were excluded a priori.

### 2.12 Drug sensitivity analysis

Drug sensitivity data were obtained from the Genomics of Drug Sensitivity in Cancer (GDSC) database [23]. Melanoma cell line expression data were extracted from the Cancer Cell Line Encyclopedia (CCLE) [24]. CAF scores were calculated for each cell line, and Spearman correlation was used to assess associations with drug IC50 values (log-transformed). Drugs with p < 0.05 were considered significantly associated.

## 3 Results

### 3.1 Single-cell transcriptomic landscape of melanoma tumor microenvironment

**Fig 1:**
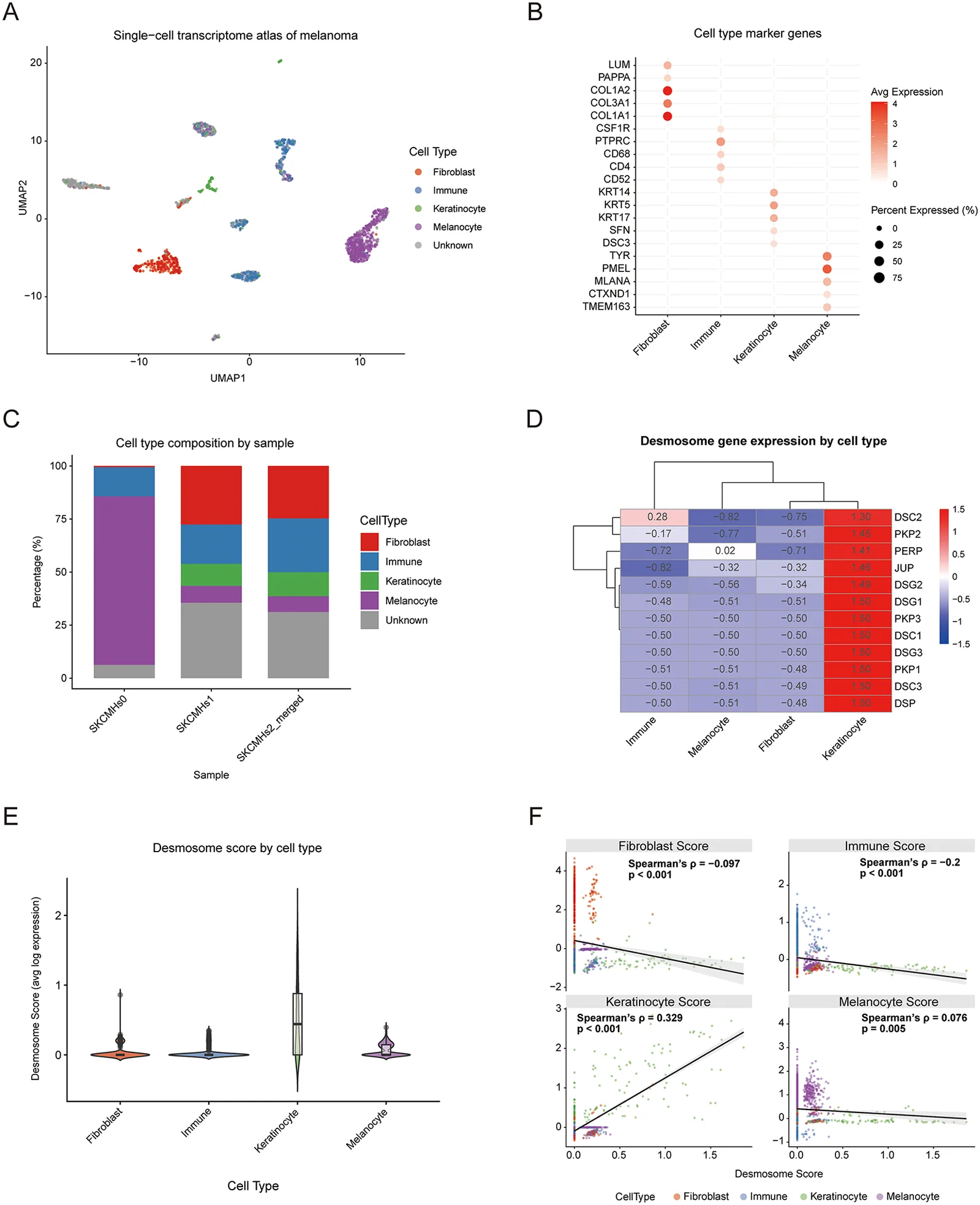
Single-cell transcriptomic landscape of melanoma tumor microenvironment. **(A)** UMAP visualization of 1,832 single cells from three primary melanoma samples. Cells are colored by annotated cell types: Fibroblast, Immune, Keratinocyte, Melanocyte, and Unknown. **(B)** Bubble heatmap showing the expression of top 5 marker genes for each annotated cell type. Dot size represents the percentage of cells expressing the gene, and color intensity represents the average expression level. **(C)** Stacked bar plot showing the cellular composition of each melanoma sample (SKCMHs0, SKCMHs1, SKCMHs2_merged). Percentages indicate the proportion of each cell type within each sample. **(D)** Heatmap displaying the average expression levels of 12 desmosome-related genes across the four major cell types (excluding Unknown). Expression values are row-scaled for visualization. **(E)** Violin plot showing the distribution of desmosome module score (average log-expression of the 12 desmosome genes) across cell types. *** indicates p < 0.001 by Wilcoxon rank-sum test (keratinocytes vs. melanocytes). **(F)** Scatter plots showing the correlation between desmosome module score and cell type-specific module scores for the four major cell types. Each point represents an individual cell. Spearman correlation coefficients (ρ) and corresponding p-values are indicated in each panel. Shaded areas represent 95% confidence intervals of the linear regression fit.

To characterize the cellular composition of melanoma tumors at single-cell resolution, we performed scRNA-seq on three primary melanoma samples (SKCMHs0, SKCMHs1, and SKCMHs2). After quality control and filtering, a total of 1,832 high-quality cells were retained for downstream analysis **(Supplementary Fig 1A-D** in S1 File). Unsupervised clustering followed by UMAP visualization revealed five distinct cell populations based on canonical marker gene expression (Fig 1A). These populations were annotated as melanocytes (TYR, PMEL, MLANA), keratinocytes (KRT14, KRT5, KRT17), immune cells (PTPRC, CD68, CD4), fibroblasts (COL1A2, DCN, LUM), and an unclassified group (Unknown) comprised of cells with low module scores (Fig 1B). Sample-to-sample expression correlation confirmed high consistency between samples (Pearson correlation > 0.77; **Supplementary Fig 1E** in S1 File).

The cellular composition varied considerably across samples (Fig 1C). Melanocytes were the predominant cell type in SKCMHs0 (79.4%), while SKCMHs1 and SKCMHs2_merged exhibited higher proportions of fibroblasts (27.6% and 24.8%, respectively) and immune cells (18.6% and 25.4%, respectively). Keratinocytes were exclusively detected in SKCMHs1 (10.4%) and SKCMHs2_merged (11.3%), consistent with the presence of epidermal components in these tumor specimens.

Given the central role of desmosomes in cell-cell adhesion and their frequent mutations in melanoma, we examined the expression pattern of 12 desmosome-related genes (DSC1–3, DSG1–3, DSP, PKP1–3, JUP, PERP) across different cell types (Fig 1D). Notably, these genes exhibited markedly higher expression in keratinocytes, particularly DSP (desmoplakin), PERP, DSC3, and JUP, whereas their expression was minimal in other cell types. Consistently, a desmosome module score calculated as the average expression of these 12 genes was significantly higher in keratinocytes compared to melanoma cells (Wilcoxon rank-sum test, p < 0.001), with intermediate levels observed in fibroblasts and immune cells (Fig 1E).

To further validate the cell type specificity of desmosome expression, we examined the correlation between the desmosome score and cell type-specific module scores. The desmosome score showed a significant positive correlation with the keratinocyte score (Spearman’s ρ = 0.329, p < 0.001), while negative or weak correlations were observed with immune (ρ = −0.200, p < 0.001), fibroblast (ρ = −0.097, p < 0.001), and melanocyte (ρ = 0.076, p = 0.005) scores (Fig 1F). These results collectively demonstrate that desmosome gene expression is predominantly restricted to keratinocytes within the melanoma tumor microenvironment, supporting their potential role in mediating keratinocyte-melanoma cell interactions.

### 3.2 Spatial transcriptomic landscape of melanoma tumor microenvironment

**Fig 2:**
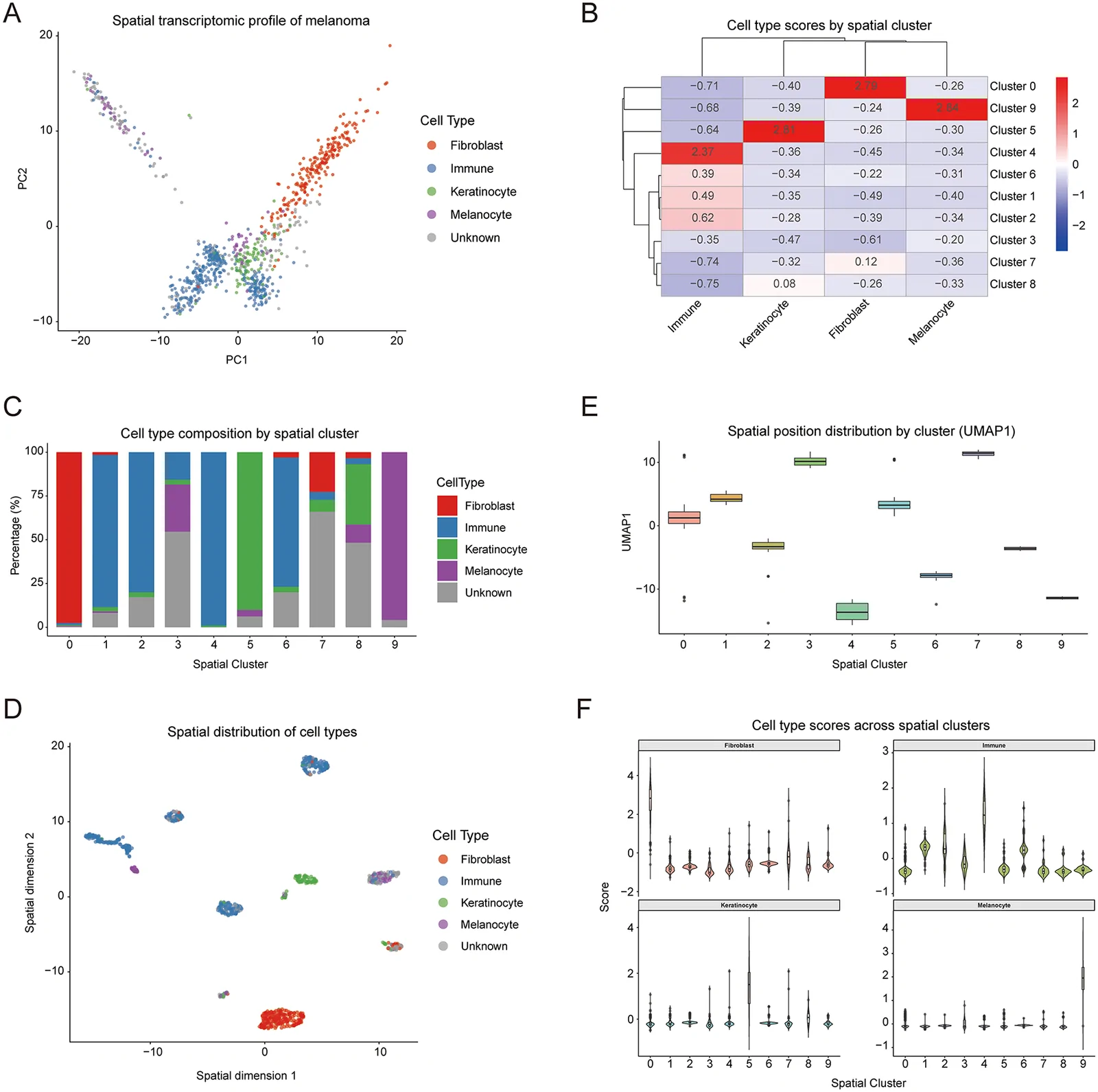
Spatial transcriptomic landscape of melanoma tumor microenvironment. **(A)** PCA of 892 ROIs colored by predicted cell type. Each point represents an individual ROI. **(B)** Heatmap showing the mean cell type scores (Fibroblast, Immune, Keratinocyte, Melanocyte) for each of the 10 spatial clusters. Colors represent scaled scores (blue: low, red: high). **(C)** Stacked bar plot showing the percentage composition of cell types within each spatial cluster. **(D)** UMAP projection of all ROIs colored by predicted cell type, visualizing the relative spatial distribution of different cell type-enriched regions. **(E)** Boxplot showing the spatial positions (UMAP1 coordinates) of ROIs across clusters. *** indicates p < 0.001 for comparison between Cluster 0 and Cluster 9 (Wilcoxon rank-sum test). **(F)** Violin plots displaying the distribution of cell type-specific scores across the 10 spatial clusters. Each panel represents one cell type score.

To investigate the spatial organization of cell types within the melanoma microenvironment, we performed GeoMx digital spatial profiling on primary melanoma samples, generating transcriptomic profiles for 892 ROIs. Quality control assessment revealed a median of 1,072 UMIs and 652 detected genes per ROI, with stable sequencing saturation across ROIs (median 91.7%; **Supplementary Fig 2A-C** in S1 File). UMI counts were comparable across different cell type-enriched ROIs (**Supplementary Fig 2D** in S1 File). PCA revealed distinct segregation of ROIs based on their cellular composition (Fig 2A). Fibroblast-enriched ROIs clustered predominantly on the right side of PC1, while immune- and melanocyte-enriched ROIs showed distinct distribution patterns.

Unsupervised clustering of the 892 ROIs identified 10 spatially distinct clusters (Fig 2B). Cluster 0 was characterized by high fibroblast scores (clustering with 97.6% fibroblasts), while Cluster 5 exhibited high keratinocyte scores, and Clusters 3, 7, and 9 showed enrichment for melanocyte scores (Fig 2B**-**2C). Consistent with these assignments, Cluster 0 demonstrated significantly higher fibroblast scores compared to Cluster 9 (Wilcoxon rank-sum test, p < 0.001), and Cluster 5 showed significantly higher keratinocyte scores compared to Cluster 9 (p < 0.001).

Spatial mapping of ROIs using UMAP projection revealed the relative organization of different cell type-enriched regions (Fig 2D). Fibroblast-enriched ROIs localized to one region, while immune-enriched ROIs occupied a distinct area, suggesting spatial compartmentalization of the tumor microenvironment. The spatial separation between fibroblast- and melanocyte-dominated clusters was statistically significant (p < 0.001; Fig 2E).

Violin plots further confirmed the cell type-specific scoring patterns across clusters (Fig 2F). Notably, keratinocyte scores were highly enriched in Cluster 5, consistent with the desmosome expression pattern observed at single-cell level (Fig 1D**-**1E). These data demonstrate that melanoma tumors exhibit spatially organized cellular niches, with fibroblasts, immune cells, keratinocytes, and melanoma cells occupying distinct spatial domains.

### 3.3 CAF-mediated immune exclusion barrier in melanoma microenvironment

**Fig 3:**
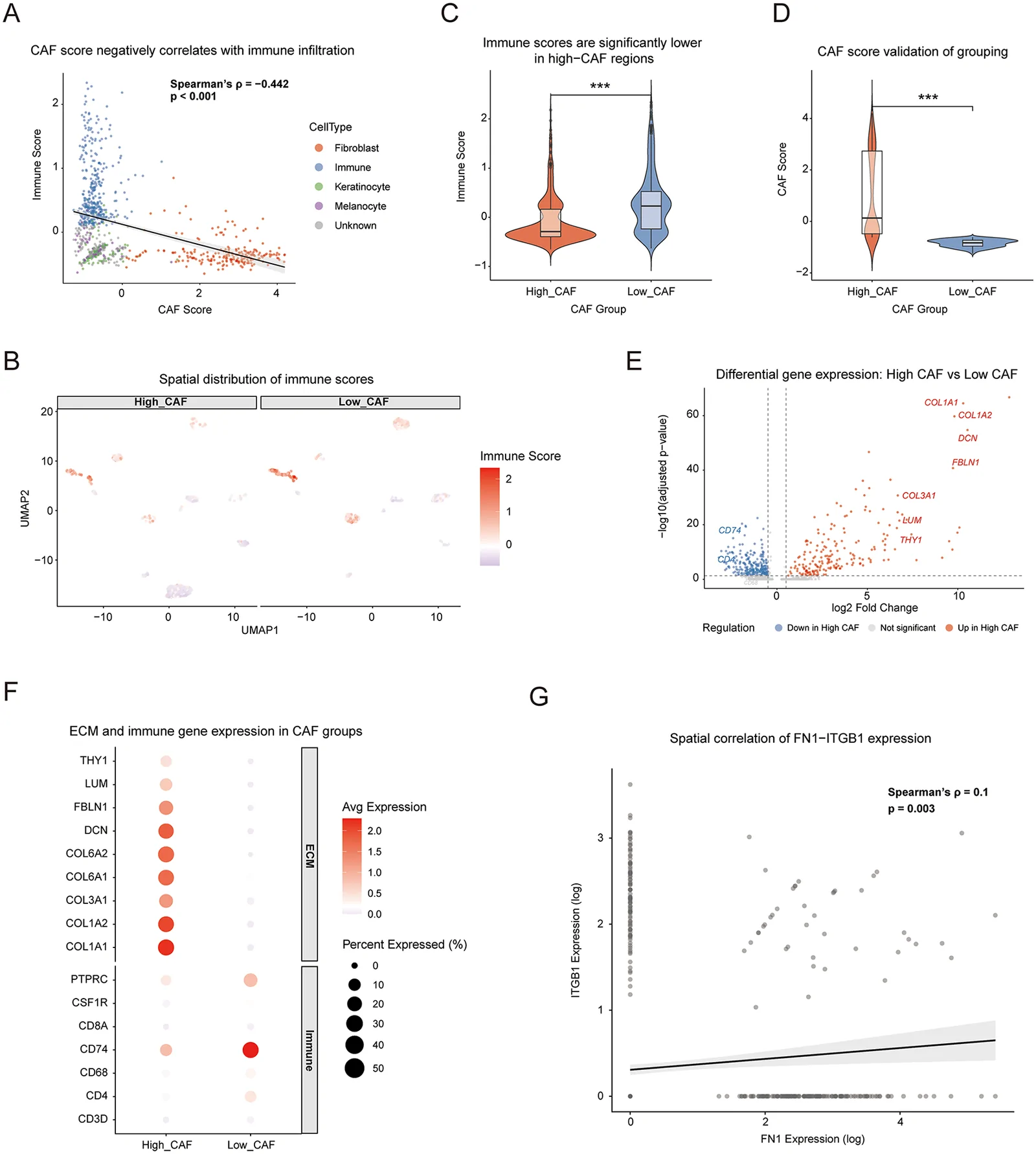
CAF-mediated immune exclusion barrier in melanoma microenvironment. **(A)** Scatter plot showing the correlation between CAF score and immune score across all 892 ROIs. Each point represents an individual ROI. Spearman correlation coefficient (ρ) and p-value are indicated. Blue line represents linear regression fit with 95% confidence interval (shaded area), shown for visualization purposes only; statistical significance was assessed using Spearman correlation. **(B)** Spatial UMAP projection of immune scores stratified by CAF status (High CAF vs Low CAF). Color intensity represents immune score (blue: low, red: high). **(C)** Violin plot comparing immune scores between High-CAF and Low-CAF regions. *** indicates p < 0.001 (Wilcoxon rank-sum test). Boxplots inside violins show median and interquartile range. **(D)** Violin plot validating CAF score differences between High-CAF and Low-CAF groups. *** indicates p < 0.001. **(E)** Volcano plot showing differential gene expression between High-CAF and Low-CAF regions. Red dots: significantly upregulated genes in High-CAF (log2FC > 0.5, adj. p < 0.05). Blue dots: significantly downregulated genes in High-CAF (log2FC < −0.5, adj. p < 0.05). Gray dots: non-significant genes. Selected ECM and immune genes are labeled. **(F)** Dot plot showing expression of key ECM (left) and immune (right) genes across CAF groups. Dot size represents percentage of cells expressing the gene; color intensity represents average expression level. **(G)** Scatter plot showing the spatial correlation between FN1 and ITGB1 expression. Each point represents an individual ROI. Spearman correlation coefficient (ρ) and p-value are indicated. Gray points represent all ROIs; black line represents linear regression fit with 95% confidence interval (shaded area), shown for visualization purposes only; statistical significance was assessed using Spearman correlation.

To investigate whether CAFs contribute to immune exclusion in melanoma, we first examined the spatial relationship between CAF scores and immune scores across all ROIs. The median CAF score across all ROIs was 0.15 (IQR −0.32 to 0.58); ROIs with CAF score above the median were classified as high-CAF, and those below as low-CAF. Spearman correlation analysis revealed a significant negative correlation between CAF score and immune score (ρ = −0.442, p < 0.001; Fig 3A), indicating that regions with high CAF activity are associated with reduced immune infiltration. The robustness of CAF grouping was validated using alternative CAF markers (FAP, PDPN, THY1, POSTN) and extreme quartile thresholding **(Supplementary Fig 3A-B in** S1 File**).**

Spatial mapping of immune scores stratified by CAF status clearly demonstrated this pattern (Fig 3B). Low-CAF regions exhibited widespread high immune scores (red), whereas high-CAF regions showed predominantly low immune scores (blue). Quantitative comparison confirmed that immune scores were significantly lower in high-CAF regions compared to low-CAF regions (median: −0.29 vs 0.23; Wilcoxon rank-sum test, p = 1.43e-29; Fig 3C). The CAF grouping was validated by the expected significant difference in CAF scores between groups (p < 0.001; Fig 3D).

To characterize the molecular basis of this immune exclusion, we performed differential gene expression analysis between high-CAF and low-CAF regions (Fig 3E). A total of 192 genes were significantly upregulated in high-CAF regions, including ECM-related genes such as COL1A1, COL1A2, COL6A2, DCN, and FBLN1. Conversely, 241 genes were downregulated in high-CAF regions, predominantly immune-related genes including PTPRC (CD45), CD68, CD4, CD8A, and CSF1R. Dot plot visualization confirmed that ECM genes (COL1A1, COL1A2, DCN, LUM, THY1) were highly expressed in high-CAF regions, while immune genes (PTPRC, CD68, CD4, CD8A, CD74, HLA-DRA) were enriched in low-CAF regions (Fig 3F). The differential expression of ECM core genes was further validated in boxplot analyses **(Supplementary Fig 3C in S1 File).**

Finally, we examined the spatial co-expression of the ECM ligand FN1 and its integrin receptor subunit ITGB1. Spearman correlation analysis revealed a significant positive correlation between FN1 and ITGB1 expression across all ROIs (ρ = 0.100, p = 0.003; Fig 3G). This supported the presence of FN1-integrin signaling interactions within the tumor microenvironment. We also examined other ECM-receptor pairs. Spearman correlation analysis revealed that COL1A1-ITGA1 showed a stronger correlation (ρ = 0.192, p = 7.4 × 10^-9^), while COL1A2-ITGA2 showed no significant positive correlation (ρ = −0.081, p = 0.016). These results suggest that the ECM-receptor signaling observed in our data may be predominantly mediated by collagen-integrin interactions rather than FN1-ITGB1 alone, highlighting the complexity of ECM-immune crosstalk.

Taken together, these findings suggest that CAFs may contribute to an immune-excluded microenvironment through ECM remodeling, with evidence of spatial co-expression of ECM-receptor pairs such as FN1-ITGB1.

### 3.4 Spatial compartmentalization of melanoma cell states

**Fig 4:**
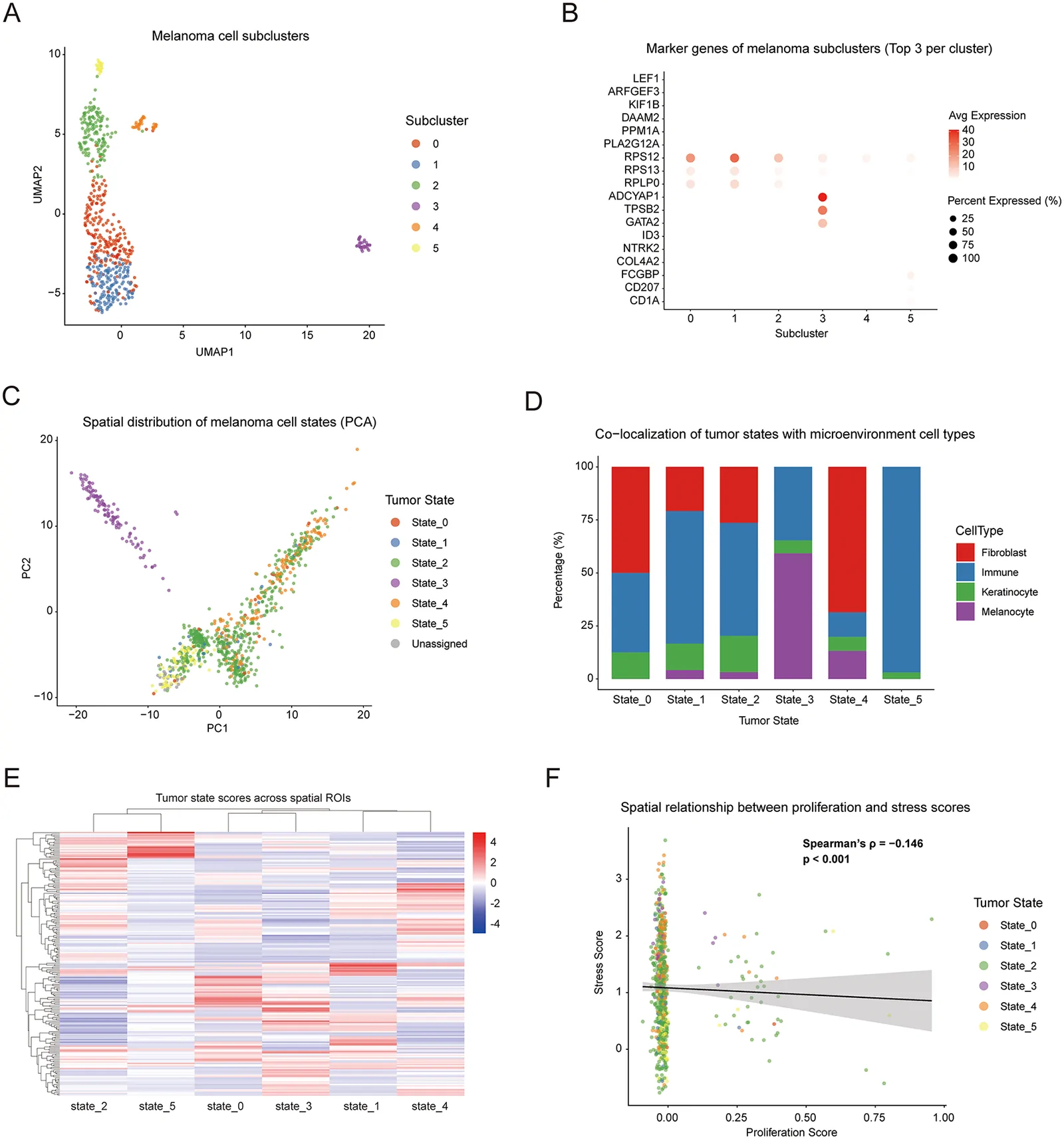
Spatial compartmentalization of melanoma cell states. **(A)** UMAP visualization of 588 melanoma cells subclustered into 6 distinct states (State 0–5). Each point represents an individual cell. **(B)** Bubble heatmap showing the top 3 marker genes for each melanoma subcluster. Dot size represents the percentage of cells expressing each gene; color intensity represents the average expression level. **(C)** Spatial projection of tumor states onto PCA coordinates derived from spatial transcriptomic data. Each point represents an individual ROI colored by the assigned tumor state. Percentages indicate the proportion of each tumor state among tumor-associated ROIs. **(D)** Stacked bar plot showing the co-localization percentages between each tumor state and microenvironment cell types (Fibroblast, Immune, Keratinocyte, Melanocyte). **(E)** Heatmap displaying tumor state module scores across spatial ROIs. Rows represent individual ROIs (n = 200 shown), columns represent the six tumor states. Color intensity indicates scaled module score (blue: low, red: high). **(F)** Scatter plot showing the correlation between proliferation score and stress score across all tumor-associated ROIs. Each point represents an individual ROI, colored by tumor state. Spearman correlation coefficient (ρ) and p-value are indicated. The boxplot inset compares proliferation scores between State 4 (fibroblast-associated) and State 5 (immune-associated). ** indicates p < 0.01 (Wilcoxon rank-sum test).

To investigate the functional heterogeneity of melanoma cells and their spatial organization within the tumor microenvironment, we first performed subclustering of 588 melanoma cells identified from the single-cell dataset (Fig 4A). Unsupervised clustering resolved six distinct melanoma subclusters (State 0–5), each characterized by unique marker gene expression profiles (Fig 4A**–**4B). State 0 expressed LEF1 and CTNNB1, suggestive of Wnt/β-catenin signaling activation. State 1 expressed proliferation-associated genes including MCM4 and DAAM2. State 2, the largest subcluster (n = 136), expressed a mixed signature. State 3 showed expression of pigmentation genes, while State 4 and State 5 exhibited distinct transcriptional programs.

We next mapped these tumor cell states onto spatial transcriptomic data by calculating module scores based on the top 10 marker genes of each state (Fig 4C). Spatial projections revealed that different tumor states occupied distinct spatial niches (Fig 4C). Notably, State 2 was the most prevalent, accounting for 55.9% of tumor-associated ROIs, whereas State 0 and State 1 were relatively rare (2.4% and 2.8%, respectively).

Analysis of co-localization between tumor states and microenvironment cell types revealed striking spatial associations (Fig 4D). State 4 showed strong co-localization with fibroblasts (68.6% of State 4 ROIs), while State 5 exhibited predominant co-localization with immune cells (96.9%). In contrast, State 3 was primarily associated with melanoma cell-enriched regions (59.2%), and State 0 displayed a mixed pattern with fibroblast (50.0%) and immune cell (37.5%) co-localization. These findings demonstrate that different melanoma cell states are spatially compartmentalized in association with distinct microenvironmental niches.

To further characterize the functional properties of each tumor state, we visualized the spatial distribution of their module scores across spatial ROIs (Fig 4E). The heatmap confirmed that each state exhibited highest scores in its corresponding ROIs, validating the robustness of the spatial mapping.

Finally, we investigated the spatial relationship between proliferation and cellular stress responses. Spearman correlation analysis revealed a significant but weak negative correlation between proliferation score and stress score (ρ = −0.146, p < 0.001; Fig 4F). Notably, comparison between tumor states with opposing microenvironment associations revealed that State 4 (fibroblast-associated) exhibited significantly higher proliferation scores compared to State 5 (immune-associated) (Wilcoxon rank-sum test, p = 0.007; Fig 4F inset), suggesting that CAF-rich microenvironments may support a more proliferative tumor state. The robustness of the proliferation and stress scores was validated through correlation with single-gene markers (MKI67 for proliferation, HSPA1A for stress) and through sensitivity analysis using alternative gene sets **(Supplementary Fig 4A-C** in S1 File**).**

Taken together, these data demonstrate that melanoma tumors exhibit pronounced spatial compartmentalization of distinct tumor cell states, with varying associations with CAF-rich, immune-rich, and tumor-rich niches, and that CAF-associated tumor states display elevated proliferative capacity.

### 3.5 TCGA complementary analysis of CAF-associated features in melanoma

**Fig 5:**
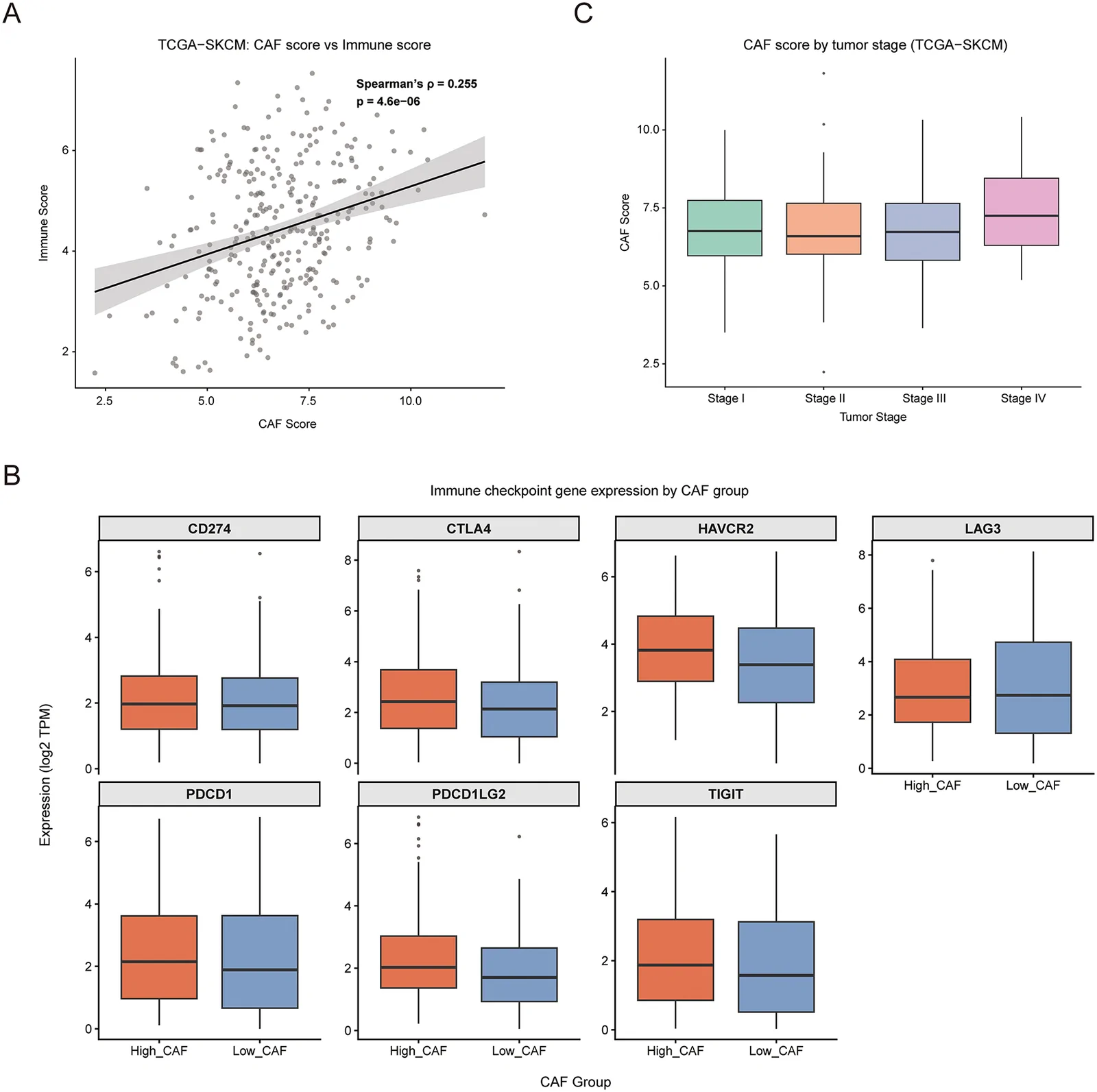
TCGA validation of CAF-associated features in melanoma. **(A)** Scatter plot showing the correlation between CAF score and immune score in 316 TCGA-SKCM samples. Each point represents an individual tumor sample. Spearman correlation coefficient (ρ) and p-value are indicated. Blue line represents linear regression fit with 95% confidence interval (shaded area), shown for visualization purposes only; statistical significance was assessed using Spearman correlation. **(B)** Boxplots showing expression levels of immune checkpoint genes in high-CAF (red) vs low-CAF (blue) groups. ** indicates p < 0.01 (Wilcoxon rank-sum test). HAVCR2 (TIM-3) and PDCD1LG2 (PD-L2) showed significantly higher expression in high-CAF tumors. **(C)** Boxplot showing CAF score distribution across tumor pathological stages (Stage I to IV). No significant difference was observed (Kruskal-Wallis test, p = 0.395). Boxes represent median and interquartile range; whiskers indicate 1.5 × IQR.

To examine the broader relevance of our spatial findings in an independent cohort, we analyzed bulk RNA-seq data from 316 TCGA-SKCM samples. The cohort included 104 primary and 212 metastatic melanoma samples. Median patient age was 58 years (IQR 48–68), with 58% male and 42% female. Tumor stage distribution was: Stage I (12%), Stage II (28%), Stage III (35%), and Stage IV (25%). Consistent with the spatial observation that CAF-rich regions exhibit altered immune profiles, we first examined the relationship between CAF score and overall immune score. Spearman correlation analysis revealed a significant positive correlation between CAF score and immune score (ρ = 0.255, p = 4.6 × 10^-6^; Fig 5A), indicating that tumors with higher CAF activity are associated with increased global immune infiltration at the bulk level.

We next evaluated the expression of key immune checkpoint genes between high-CAF and low-CAF groups (Fig 5B). While most checkpoint genes (PDCD1, CTLA4, LAG3, TIGIT, CD274) showed no significant differences, HAVCR2 (TIM-3) and PDCD1LG2 (PD-L2) were significantly upregulated in high-CAF tumors (HAVCR2: p = 0.0022, fold change = 1.13; PDCD1LG2: p = 0.0032, fold change = 1.19), suggesting potential CAF-associated immune modulation.

Finally, we examined whether CAF score correlated with tumor stage. No significant association was observed between CAF score and pathological stage (Kruskal-Wallis test, p = 0.395; Fig 5C), with median CAF scores remaining comparable across stages (Stage I: 6.76, Stage II: 6.59, Stage III: 6.73, Stage IV: 7.25).

Together, these TCGA analyses confirm the clinical relevance of CAF-associated immune features and identify specific immune checkpoint molecules potentially involved in CAF-mediated immunomodulation, while suggesting that CAF activation is not simply a late-stage phenomenon.

### 3.6 CAF score predicts drug sensitivity and immune features in melanoma

**Fig 6:**
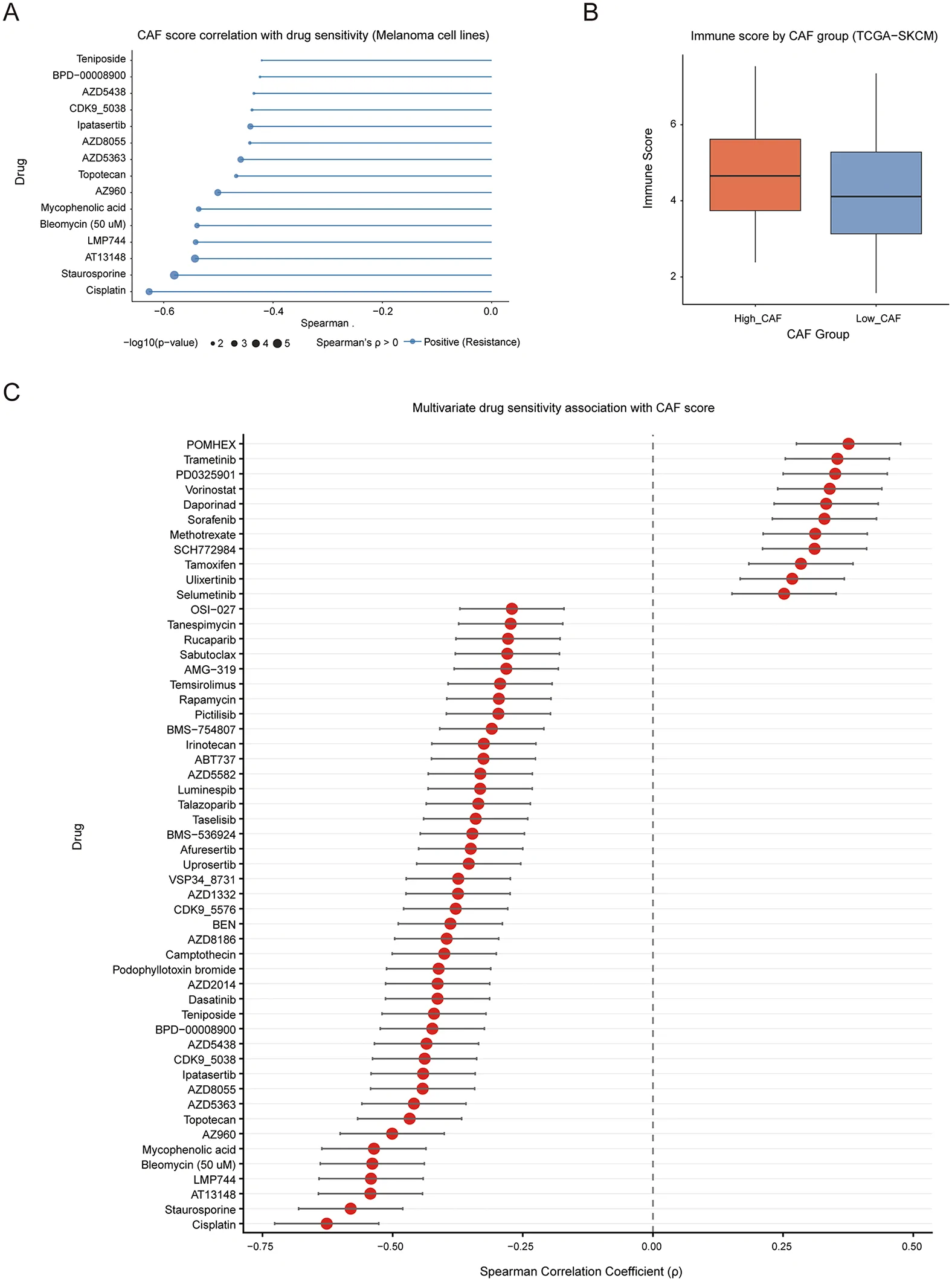
CAF score predicts drug sensitivity and immune features in melanoma. **(A)** Lollipop plot showing the correlation between CAF score and drug sensitivity (IC50) in melanoma cell lines from the GDSC database. Top 15 significantly correlated drugs are displayed. Points are colored by correlation direction (blue: negative correlation, indicating sensitivity; red: positive correlation, indicating resistance). Point size represents -log10(p-value). Spearman correlation coefficients (ρ) and p-values are indicated. **(B)** Boxplot comparing immune scores between high-CAF and low-CAF groups in the TCGA-SKCM cohort. High-CAF tumors showed significantly higher immune scores. *** indicates p < 0.001 (Wilcoxon rank-sum test). Boxes represent median and interquartile range; whiskers indicate 1.5 × IQR. **(C)** Forest plot summarizing the distribution of significant drug-CAF associations. Each point represents a drug with significant correlation (p < 0.05). Negative correlations (left side) indicate CAF-high cells are more sensitive to the drug; positive correlations (right side) indicate resistance. Error bars represent 95% confidence intervals.

To explore the clinical translational potential of the CAF signature, we first examined its association with drug sensitivity in melanoma cell lines using the GDSC database. Spearman correlation analysis revealed that CAF score was significantly associated with sensitivity to multiple drugs (Fig 6A). Notably, cisplatin showed the strongest negative correlation (ρ = −0.627, p < 0.001), indicating that cell lines with higher CAF scores were more sensitive to this agent. Other top-ranked drugs included staurosporine (ρ = −0.581, p < 0.001) and AT13148 (ρ = −0.543, p < 0.001). Conversely, a few drugs showed positive correlations, including POMHEX (ρ = 0.376, p = 0.038) and trametinib (ρ = 0.354, p = 0.009), suggesting potential resistance in CAF-high tumors.

We next evaluated the relationship between CAF score and immune features relevant to immunotherapy response. High-CAF tumors exhibited significantly higher immune scores compared to low-CAF tumors (median: 4.66 vs 4.11, Wilcoxon rank-sum test, p = 0.0008; Fig 6B), suggesting that CAF-high tumors may possess a more inflamed tumor microenvironment.

Finally, we performed a comprehensive assessment of drug sensitivity associations. Among 53 drugs with significant correlations (p < 0.05), 42 showed negative correlations (sensitivity in CAF-high cells) and 11 showed positive correlations (resistance in CAF-high cells) (Fig 6C). Cisplatin remained the most strongly associated agent.

Collectively, these findings suggest that the CAF signature is correlated with drug sensitivity in melanoma cell lines. However, because CAFs are absent from conventional cell line models, these findings should be considered hypothesis-generating. Further investigation in CAF-containing experimental systems is needed to evaluate the clinical relevance of these correlations.

## 4 Discussion

In this study, we integrated scRNA-seq and spatial transcriptomics to investigate the role of CAFs in shaping the melanoma tumor microenvironment. Our analysis identified three major findings. First, we identified a strong negative correlation between CAF scores and immune scores across 892 spatial ROIs, suggesting spatially resolved CAF-mediated immune exclusion. Second, differential gene expression analysis showed that CAF-high regions are enriched for ECM-related genes, including *COL1A1*, *COL1A2*, *DCN*, and *FBLN1*, while immune-related genes such as *PTPRC*, *CD4*, and *CD8A* are downregulated, with spatial co-expression of FN1 and its receptor ITGB1 supporting a role for ECM-receptor signaling in this process. Third, we observed spatial compartmentalization of six distinct melanoma cell states, with CAF-associated tumor states exhibiting significantly higher proliferative capacity than immune-associated states. Finally, we assessed the clinical relevance of the CAF signature using TCGA bulk RNA-seq data and demonstrated its potential utility in predicting drug sensitivity, including sensitivity to cisplatin and resistance to trametinib.

The physical exclusion of T cells from the tumor core is a well-recognized mechanism of immunotherapy resistance [5–7]. Our spatial transcriptomic data show that CAF-rich regions in melanoma are associated with significantly lower immune scores, primarily driven by reduced T cell infiltration. This finding aligns with recent studies demonstrating that CAFs, particularly the matrix CAF (mCAF) subtype, deposit dense collagen networks that act as physical barriers to T cell migration [5,9,11]. In non-small cell lung cancer, similar observations have shown that CAF-expressed *COL11A1* and *COL4A1* are associated with T cell exclusion. Our observation that ECM genes, including *COL1A1*, *COL1A2*, and *FBLN1*, are specifically upregulated in CAF-high regions extends these findings to melanoma. Notably, FBLN1, a glycoprotein involved in ECM organization, has recently been identified as a CAF-associated signature gene in melanoma [3,25]. The spatial co-expression of FN1 and its receptor ITGB1 in our data further suggests that FN1-ITGB1 signaling may play a role in CAF-ECM-immune crosstalk, potentially by promoting matrix stiffness and integrin-mediated T cell retention in the stroma [6,13,15]. We acknowledge that an alternative interpretation of this observation is spatial compartmentalization—the segregation of CAF-rich and immune-rich regions may reflect tissue architecture rather than active CAF-mediated exclusion. However, the enrichment of ECM-related genes in CAF-high regions and the spatial co-expression of FN1-ITGB1 are consistent with a potential functional role for CAFs in creating a matrix-associated barrier. Nonetheless, our correlative data cannot distinguish between these possibilities, and functional experiments are needed to establish causality. We acknowledge that our pan-CAF score may have diluted the FN1 signal, as FN1 is predominantly expressed by mCAFs rather than iCAFs. An mCAF-specific analysis could potentially yield stronger correlations with ECM-receptor signaling. We therefore examined additional ECM-receptor pairs, including COL1A1-ITGA1 and COL1A2-ITGA2. Interestingly, COL1A1-ITGA1 showed a stronger correlation (ρ = 0.192, p = 7.4 × 10^-9^) than FN1-ITGB1 (ρ = 0.100, p = 0.003), while COL1A2-ITGA2 showed no significant positive correlation (ρ = −0.081, p = 0.016). These results suggest that collagen-integrin interactions, particularly involving COL1A1, may also contribute to the ECM-immune signaling axis, and support the reviewer’s suggestion that mCAF-specific analyses could yield stronger signals. Nonetheless, FN1-ITGB1 remained significantly correlated, supporting its relevance as an ECM-receptor pair in our dataset. Future studies employing mCAF-specific signatures may provide additional resolution.

An unexpected finding of our study was the spatial segregation of six distinct melanoma cell states within the tumor microenvironment. State 4 was strongly associated with fibroblast-rich regions (68.6% co-localization) and exhibited elevated proliferative capacity compared to State 5, which co-localized with immune-rich regions (96.9%). This spatial organization suggests that the local microenvironment actively shapes melanoma cell phenotype and behavior. Prior studies have described transcriptional heterogeneity in melanoma cells, with proliferative and invasive states representing a spectrum of cellular plasticity [3,26]. Our results extend these observations by providing spatial evidence that CAF-rich niches may support a more proliferative tumor state, whereas immune-rich niches may impose selective pressure favoring less proliferative or more immune-evasive states [5,9,16]. The identification of spatially distinct tumor states within the same tumor has important therapeutic implications, as different regions may exhibit differential sensitivity to targeted therapies and immunotherapies [4,12]. These observations suggest a model of spatial compartmentalization of melanoma cell states. In this model, the local microenvironment—particularly CAF-rich versus immune-rich niches—may differentially regulate tumor cell proliferation.

The translational potential of our CAF signature is supported by its association with drug sensitivity in melanoma cell lines. The negative correlation between CAF score and cisplatin sensitivity (ρ = −0.627, p < 0.001) suggests that CAF-high tumors may be particularly susceptible to platinum-based chemotherapy. This finding is consistent with reports that CAF-rich tumors often exhibit increased sensitivity to DNA-damaging agents, potentially due to CAF-mediated modulation of the redox environment or delivery of pro-apoptotic signals [14,27,28]. Conversely, the positive correlation with trametinib (ρ = 0.354, p = 0.009) suggests that CAF-high tumors may be relatively resistant to MEK inhibition, which aligns with studies showing that CAFs can activate alternative survival pathways, including PI3K-AKT signaling, to circumvent MAPK pathway blockade [4,12]. The association between CAF scores and immune scores in TCGA bulk data (ρ = 0.255, p = 4.6 × 10^-6^), while directionally opposite to our spatial findings, likely reflects the inherent limitation of bulk RNA-seq in averaging signals across heterogeneous tissue compartments, thereby masking the spatial segregation observed at single-ROI resolution [8,29]. This apparent discordance underscores the complementary value of spatial and bulk transcriptomic approaches. This discordance does not undermine the spatial observations but rather highlights that bulk RNA-seq averages signals across tumor and stromal compartments, potentially cancelling out spatially opposed signals. Importantly, the positive correlation at the bulk level may reflect global tumor inflammation rather than local CAF-immune interactions. Collectively, these findings suggest that spatial resolution is essential for dissecting CAF-immune relationships, and that bulk-level correlations should not be interpreted as contradicting spatial observations. We acknowledge that we did not examine BRAF V600E or other mutation status in the GDSC or TCGA datasets. This analysis was intended as an exploratory screening to identify potential associations between the CAF signature and drug response, not as a definitive predictor of clinical outcome. Future studies should assess whether mutation status confounds or modifies these associations.

Recent pan-cancer spatial transcriptomic studies have identified conserved CAF subtypes with distinct spatial distributions and functional roles, including four spatial subtypes (s1–s4) across eight cancer types and four functional subtypes (iCAF, mCAF, metabolic CAF, and proliferative CAF) across six cancer types [9,16]. In melanoma, CAFs have been shown to construct immune exclusion networks via COLLAGEN-integrin signaling [15], and similar collagen-rich barriers formed by hypoxic metabolic myofibroblasts have been reported in hepatocellular carcinoma [11]. Our CAF score, based on the average expression of fibroblast-associated genes (COL1A2, DCN, COL6A2, COL1A1, FBLN1), captures features most closely aligned with the mCAF subtype. The negative correlation between CAF score and immune score in our spatial data is consistent with the established role of mCAFs in creating physical barriers that exclude T cells [5,6]. Our finding that CAF score did not significantly correlate with tumor stage in TCGA data (p = 0.395) suggests that the ECM-remodeling function of CAFs may be an early and persistent feature of melanoma progression rather than a late-stage phenomenon. We acknowledge that our pan-CAF score, based on ECM-related genes, is most closely aligned with the mCAF subtype. Therefore, the observed associations may be specific to mCAFs rather than CAFs as a whole, and future studies using subtype-specific signatures may provide additional resolution.

The identification of CAFs as key mediators of immune exclusion in melanoma has direct clinical implications. The CAF score could serve as a predictive biomarker for patient stratification, as patients with high CAF scores may be less likely to respond to immune checkpoint inhibitor monotherapy but might benefit from combination strategies targeting CAFs or ECM components [8,29,30]. The FN1-ITGB1 axis represents a potential therapeutic target, as FN1 secreted by CAFs may promote T cell adhesion to the ECM and impair their migration into tumor nests [6,15,31]. Additionally, the observation that CAF-associated tumor states exhibit higher proliferative capacity raises the question of whether disrupting CAF-ECM interactions could represent a novel therapeutic strategy [27]. Finally, our pan-CAF score aggregates all fibroblasts into a single metric, potentially masking subtype-specific contributions to drug sensitivity. Future studies using mCAF- and iCAF-specific signatures may provide additional resolution. For immunohistochemical validation of this CAF signature in routine pathology samples, we suggest using a combination of COL1A2 and DCN. COL1A2 showed the highest differential expression between CAF-high and CAF-low regions (log2FC = 1.34), and DCN is a well-established fibroblast marker with widely available commercial antibodies. Combined staining of these two markers could provide a practical approach for identifying CAF-rich regions in melanoma tissue sections.

Several limitations should be acknowledged. First, GeoMx resolution (~50–100 cells/ROI) does not achieve single-cell resolution, and ROIs may contain mixed populations that could influence CAF-immune correlation interpretation. Second, our drug sensitivity predictions are based on GDSC cell line data, which do not contain CAFs. Therefore, these findings should be interpreted with caution and require validation in CAF-containing experimental systems before any clinical interpretation can be made. Third, while Benjamini-Hochberg correction was applied for differential expression, other exploratory analyses (e.g., drug sensitivity) were not corrected for multiple testing, increasing type I error risk. Fourth, the cross-sectional nature of spatial transcriptomics precludes causal inference; reverse causation (immune-poor regions creating a niche for CAF activation) is theoretically possible. Finally, we did not examine BRAF V600E or other mutation status in the drug sensitivity analysis, which may confound the observed associations.

## 5 Conclusion

**Fig 7:**
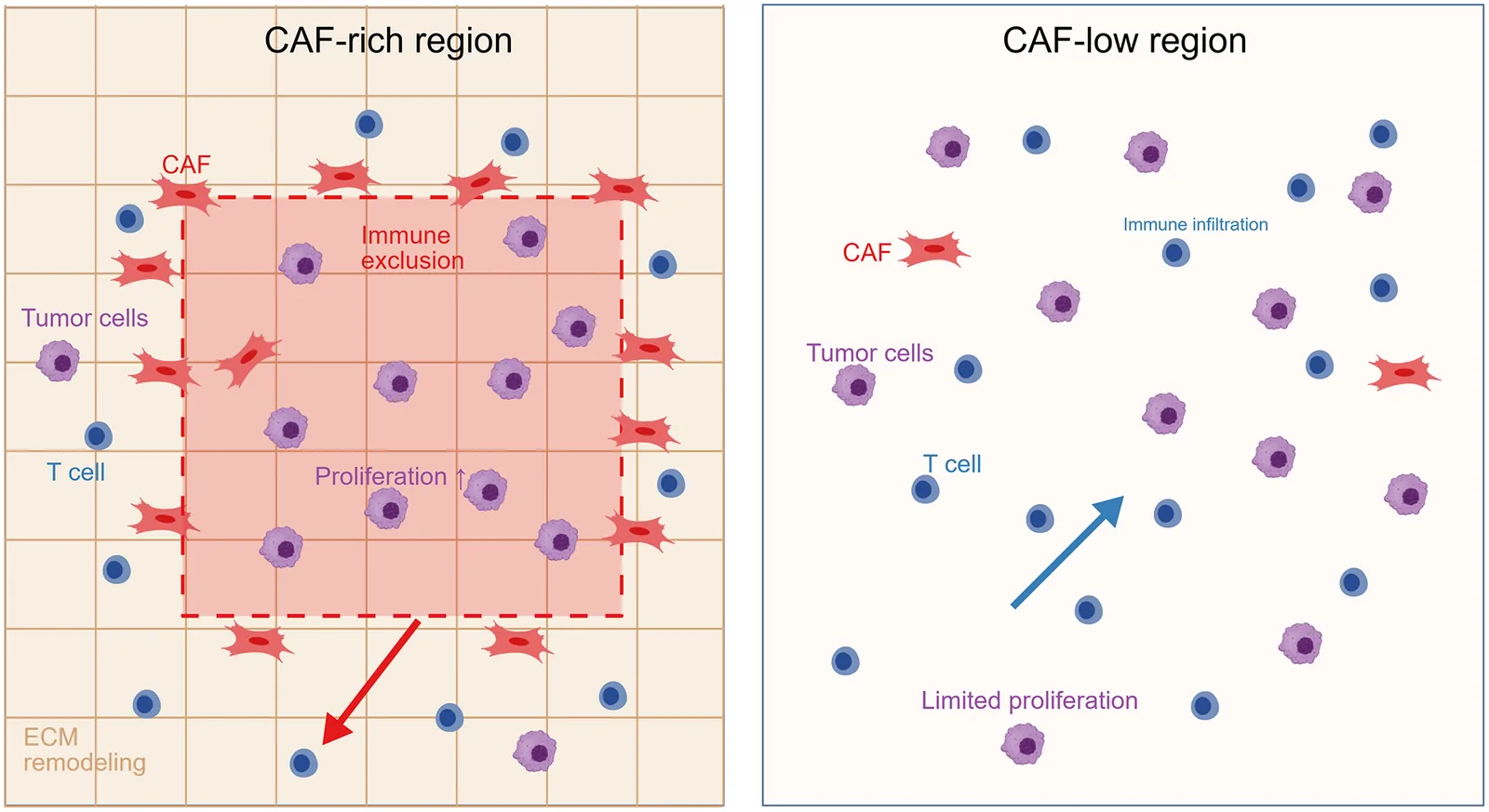
Schematic model illustrating the dual role of CAFs in shaping the melanoma tumor microenvironment. **Left panel (CAF-rich region):** In areas with high CAF density, extensive ECM remodeling creates a dense stromal network (brown mesh). CAFs (red) surround a collagen-rich barrier (dashed red border) that encloses proliferating melanoma cells (purple). This physical barrier effectively excludes T cells (blue), which remain trapped outside the barrier (red arrows). Consequently, melanoma cells within the CAF-rich niche exhibit increased proliferation (purple arrow). **Right panel (CAF-low region):** In contrast, areas with low CAF density show minimal ECM deposition and lack a physical barrier. T cells (blue) can freely infiltrate the tumor region (blue arrows), whereas melanoma cells (purple) show limited proliferative capacity.

This study provides a spatial atlas of CAF-immune interactions in melanoma by integrating single-cell and spatial transcriptomics. We show that CAF abundance is negatively correlated with immune infiltration at the spatial level, with ECM remodeling and FN1-ITGB1 signaling as potential mediators. Distinct melanoma cell states are spatially compartmentalized, with CAF-associated states exhibiting heightened proliferative capacity **(**Fig 7**)**. The CAF signature shows potential for predicting drug sensitivity (cisplatin sensitivity, trametinib resistance). We propose that spatial compartmentalization of melanoma cell states represents a previously underappreciated feature of tumor-microenvironment crosstalk that may influence therapeutic response. Validation in independent cohorts and functional studies of the FN1-ITGB1 axis are warranted.

## Supporting information

S1 File Supplementary Figs 1-4. All supplementary figures are provided in a single PDF file. (PDF)

## Abbreviations

CAFs: cancer-associated fibroblasts
ECM: extracellular matrix
scRNA-seq: single-cell RNA sequencing
ROIs: regions of interest
TCGA: The Cancer Genome Atlas
GDSC: Genomics of Drug Sensitivity in Cancer
ICIs: immune checkpoint inhibitors
mCAFs: matrix CAFs
iCAFs: immunomodulatory CAFs
PCA: principal component analysis
UMAP: uniform manifold approximation and projection
CCLE: Cancer Cell Line Encyclopedia
