# Supplementary material for: Spatial compartmentalization of melanoma cell states reveals CAF-associated tumor cells with enhanced proliferative capacity
Source: PLoS One. 2026 Sep 15;21(9):e0358235. doi: 10.1371/journal.pone.0358235 (PMC13577406; doi:10.1371/journal.pone.0358235)
Supplement: Supplementary file 1 [file pone.0358235.s001.pdf]

Supplementary figures

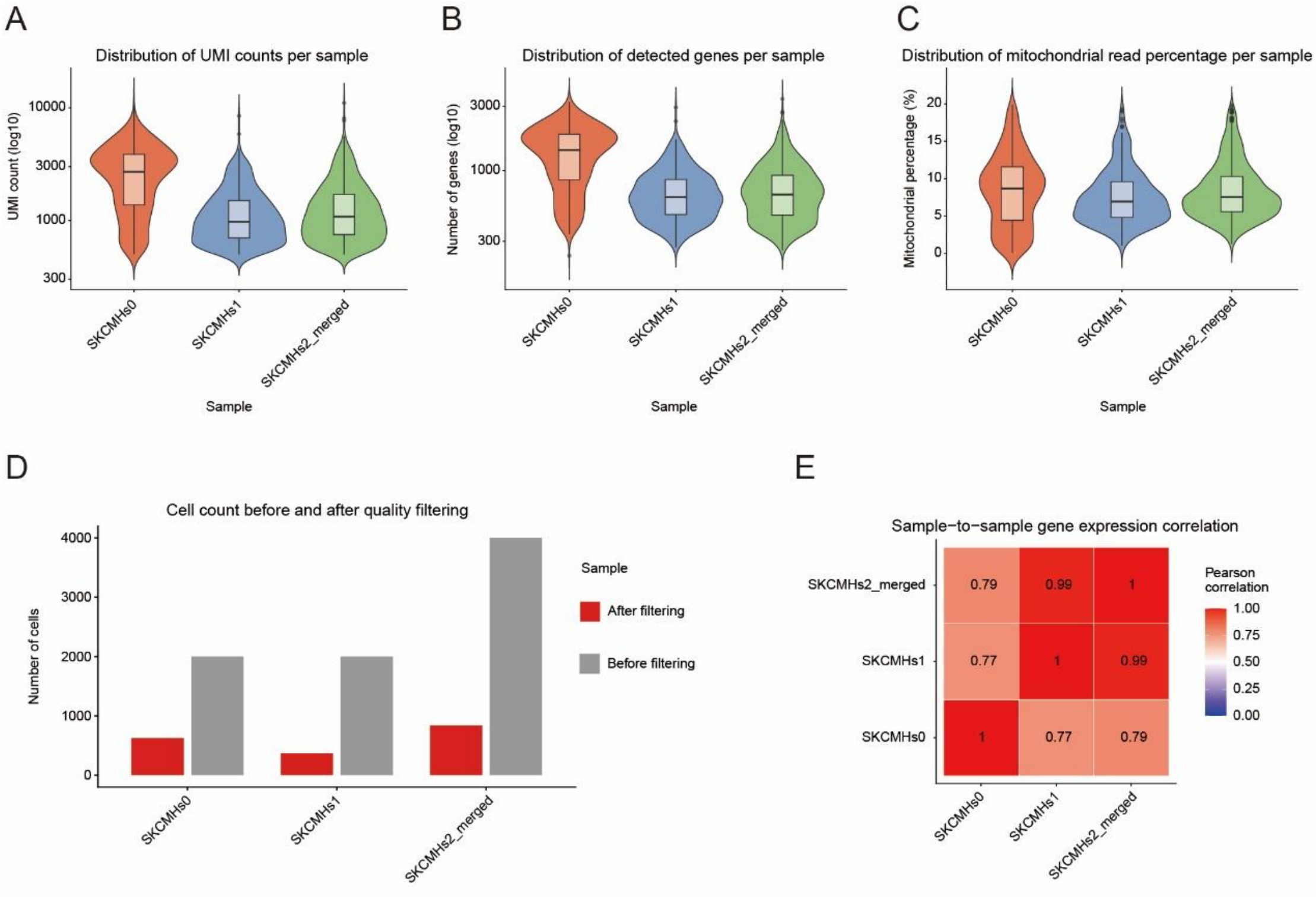

Supplementary Figure 1. Quality control of scRNA-seq data.

(A) Violin plots showing the distribution of UMI counts per cell across the three samples.

(B) Violin plots showing the distribution of detected gene numbers per cell.

(C) Violin plots showing the distribution of mitochondrial read percentages.

(D) Bar plot comparing cell numbers before and after quality filtering.

(E) Heatmap showing sample-to-sample Pearson correlation coefficients of gene expression.

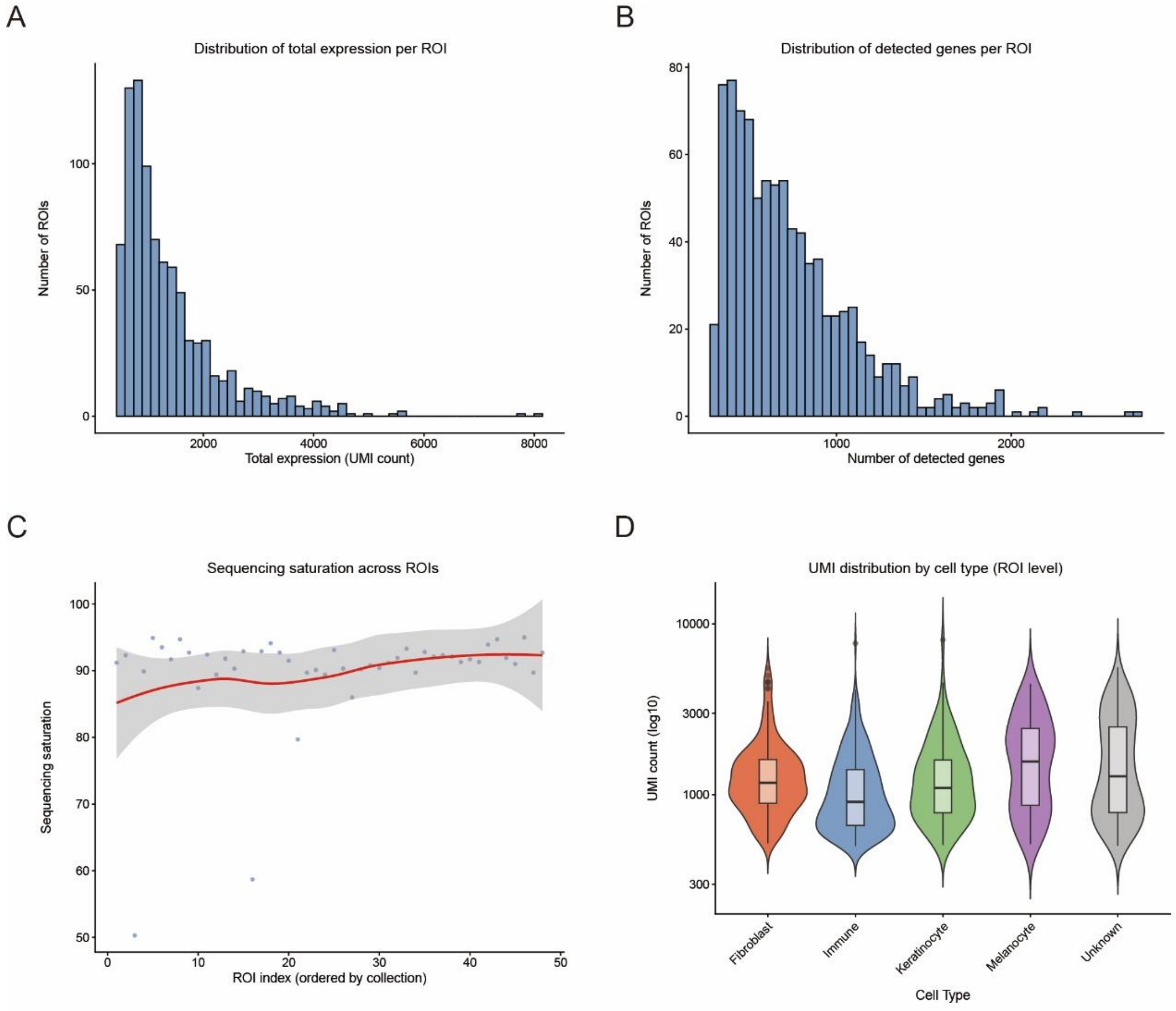

Supplementary Figure 2. Quality control of spatial transcriptomics data.

(A) Histogram showing the distribution of total UMI counts per ROI (n = 892).

(B) Histogram showing the distribution of detected gene numbers per ROI.

(C) Scatter plot showing sequencing saturation across ROIs (loess smoothing curve in red).

(D) Violin plots showing UMI distribution across different cell type-enriched ROIs.

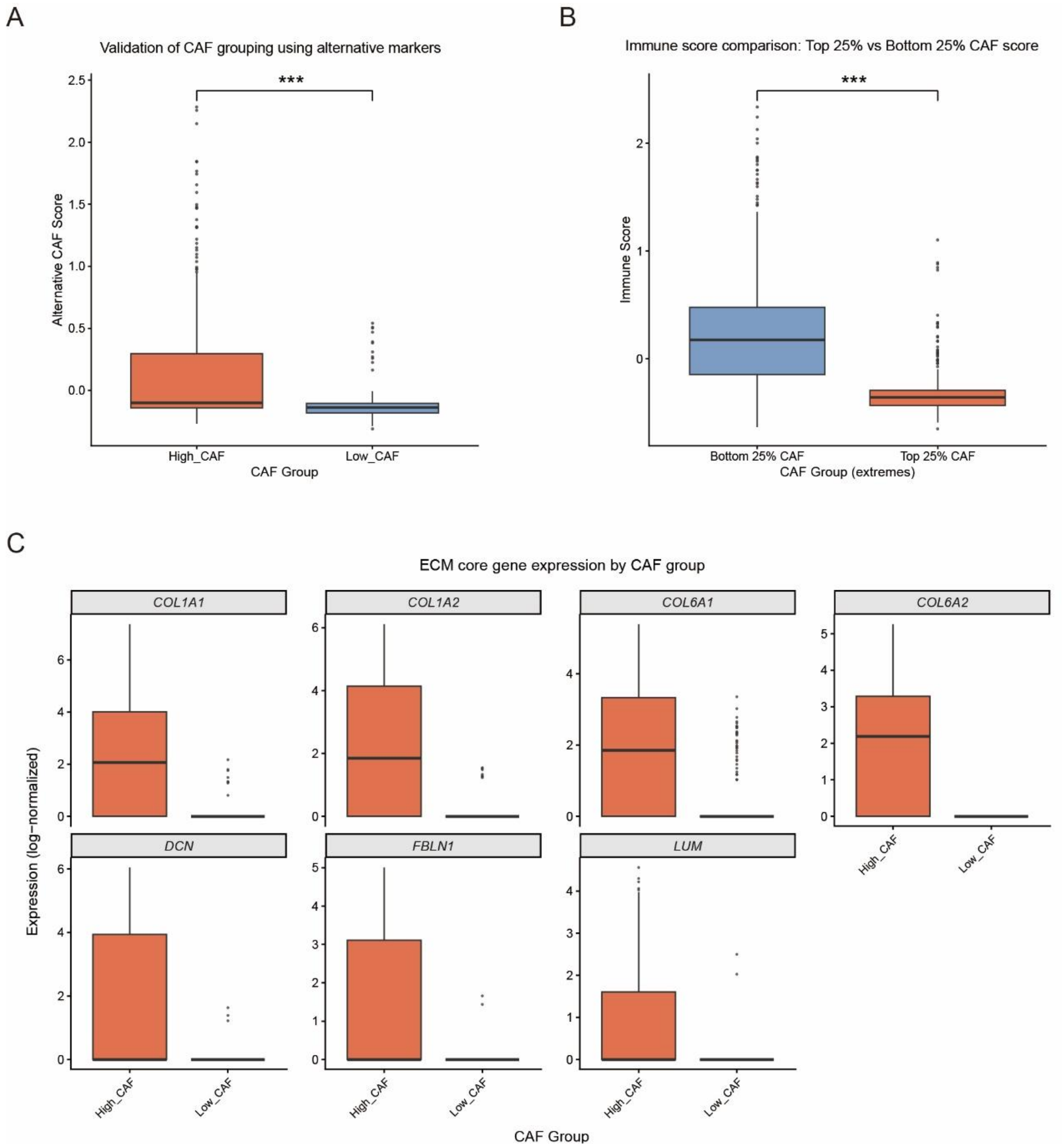

Supplementary Figure 3. Robustness validation of CAF scoring.

(A) Boxplot showing alternative CAF scores (based on FAP, PDPN, THY1, and POSTN) in high-CAF vs low-CAF groups. *** indicates $p < 0.001$ (Wilcoxon rank-sum test).

(B) Boxplot comparing immune scores between extreme CAF groups (top 25% vs bottom 25% CAF scores). *** indicates $p < 0.001$.

(C) Boxplot showing expression of ECM core genes (COL1A1, COL1A2, COL6A1, COL6A2, DCN, FBLN1, LUM) in high-CAF vs low-CAF groups. All genes showed

significantly higher expression in high-CAF regions (Wilcoxon rank-sum test, $p < 0.001$).

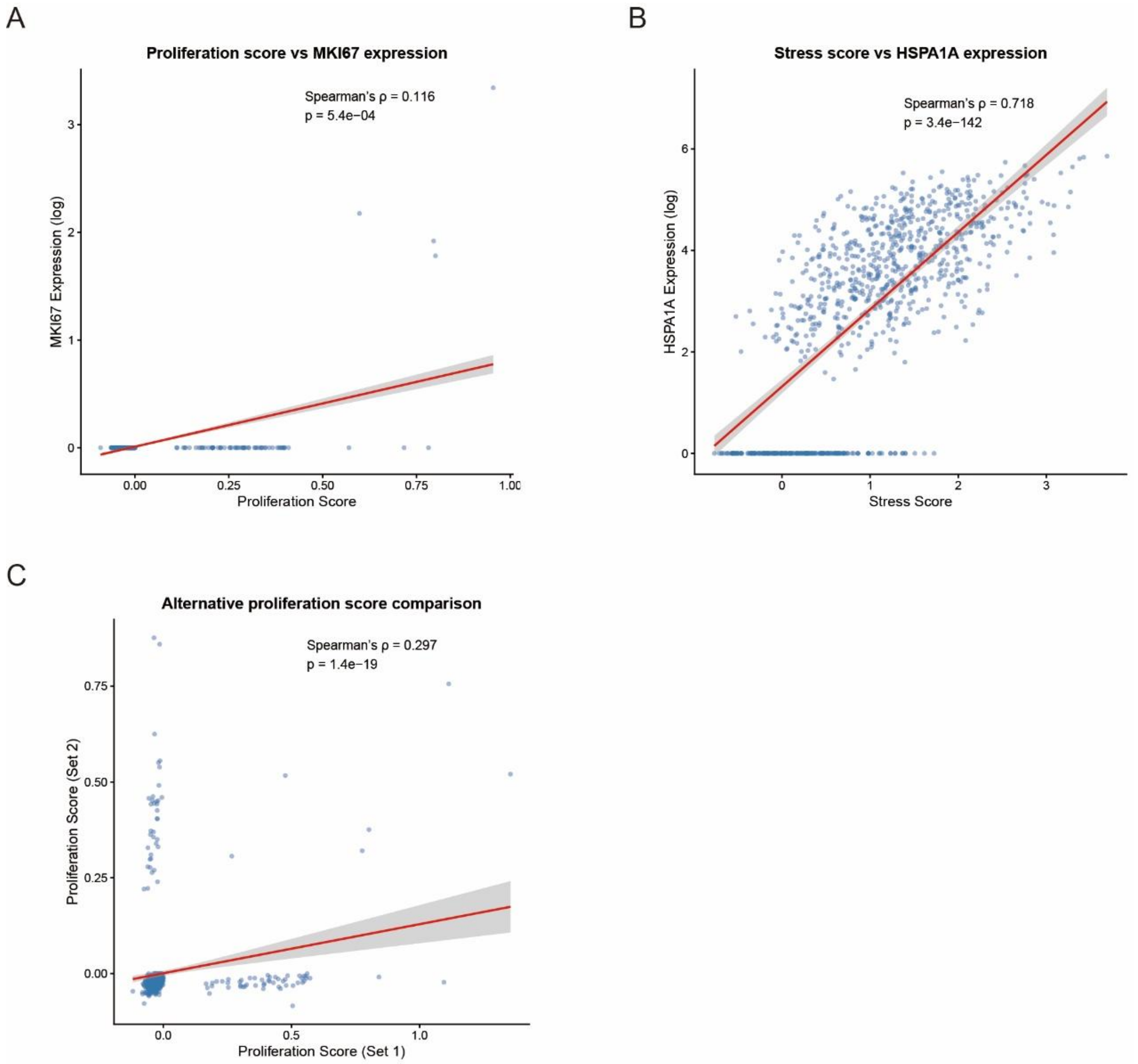

Supplementary Figure 4. Robustness validation of tumor state scores.

(A) Scatter plot showing correlation between proliferation score and MKI67 expression. Spearman correlation ρ and p-value are indicated.

(B) Scatter plot showing correlation between stress score and HSPA1A expression. Spearman correlation ρ and p-value are indicated.

(C) Scatter plot showing correlation between two independent proliferation scores calculated using different gene sets (Set 1: MKI67, PCNA, TOP2A, CDK1, CCNB1; Set 2: AURKA, BIRC5, CDK2, CCNA2, CDC20). Significant positive correlation demonstrates score robustness.
