# Supplementary material for: Spatial compartmentalization of melanoma cell states reveals CAF-associated tumor cells with enhanced proliferative capacity
Source: PLoS One. 2026 Sep 15;21(9):e0358235. doi: 10.1371/journal.pone.0358235 (PMC13577406; doi:10.1371/journal.pone.0358235)
Supplement: Supplementary file 2 [file pone.0358235.s003.docx]

Dear Dr. An and Reviewer,

We thank the editor and reviewers for their thoughtful comments and constructive suggestions on our manuscript. We have carefully considered all the points raised and have revised the manuscript accordingly. Below we provide point-by-point responses to each comment. All changes in the revised manuscript are highlighted in yellow

We believe the revised manuscript is substantially strengthened and hope it now meets the standards for publication in *PLOS ONE*.

Sincerely,

Weilong Zheng, MD
Corresponding author

Reviewer #1: This manuscript presents an integrated analysis of single-cell RNA sequencing, spatial transcriptomics, TCGA, and drug sensitivity datasets to investigate the role of cancer-associated fibroblasts (CAFs) in the melanoma tumor microenvironment. The study addresses an important topic, and the integration of multiple publicly available datasets is a strength. The manuscript is generally well organized, and the figures are clear and informative. However, several issues should be addressed before the manuscript can be considered for publication.

Major comments

The conclusions are stronger than the evidence presented. The study is entirely computational and observational. While the analyses identify associations between CAF-rich regions, immune exclusion, tumor cell states, and drug sensitivity, they do not establish causality. Statements suggesting that CAFs "create" immune exclusion or that the FN1-ITGB1 axis mediates these effects should be consistently presented as hypotheses or potential mechanisms unless supported by functional validation.

Response: We agree with the reviewer's concern. We have revised the language throughout the manuscript to ensure that all conclusions are presented as hypotheses or potential mechanisms rather than causal claims. Specifically, we have:

1. Changed "demonstrating" to "suggesting" (Discussion, first paragraph, Line 432)
2. Changed "uncovered" to "observed" (Discussion, first paragraph, Line 438)
3. Changed "provide direct evidence" to "show" (Discussion, second paragraph, Line 444)
4. Changed "demonstrate" to "show" (Conclusion, Line 573)
5. Changed "demonstrate that CAFs create" to "suggest that CAFs may contribute to" (Results 3.3) (Line 331)

Limited validation. Although TCGA data are used for validation, this represents bulk RNA-seq data and cannot validate the spatial observations directly. Moreover, the positive correlation between CAF score and immune score in TCGA contrasts with the negative correlation observed in the spatial dataset. While the authors discuss this discrepancy, additional discussion is needed to explain how this affects the biological interpretation and the robustness of the proposed model.

Response: We agree with the reviewer. We have revised our description of TCGA analysis from "validation" to "complementary analysis" throughout the manuscript (Line 11; Lines 378-379; Line 440).

We have also expanded our discussion of the discordance between spatial and bulk findings:" This discordance does not undermine the spatial observations but rather highlights that bulk RNA-seq averages signals across tumor and stromal compartments, potentially cancelling out spatially opposed signals. Importantly, the positive correlation at the bulk level may reflect global tumor inflammation rather than local CAF-immune interactions. Collectively, these findings suggest that spatial resolution is essential for dissecting CAF-immune relationships, and that bulk-level correlations should not be interpreted as contradicting spatial observations (Lines 509-516)."

Drug sensitivity analysis requires greater caution. Drug sensitivity is inferred from correlations between CAF scores and GDSC melanoma cell line data. Because CAFs are absent from conventional cell lines, it is difficult to interpret CAF-derived signatures as predictors of drug response in this context. The limitations of this analysis should be discussed more explicitly, and conclusions regarding clinical utility should be moderated.

Response: We agree with the reviewer. We have moderated the language throughout the drug sensitivity analysis and added explicit acknowledgment of the limitation that GDSC cell lines lack CAFs. Specifically, we have:

1. Revised the Abstract conclusion to reflect that these findings warrant further investigation in CAF-containing systems (Lines 26-27).
2. Revised Results 3.6 to present these findings as hypothesis-generating (Lines 423-427).
3. Added a statement in the Discussion acknowledging that cell line-based correlations may not reflect the drug response of CAF-rich tumors (Lines 509-516).
4. Added a dedicated limitation in the Limitations section stating that drug sensitivity predictions require validation in CAF-containing experimental systems (Lines 560-564).

Multiple testing. Although the manuscript states that Benjamini-Hochberg correction was applied where indicated, several exploratory analyses, including the drug sensitivity analysis, appear to rely on unadjusted p-values. The potential for false-positive findings should be discussed, and multiple-testing correction should be considered where appropriate.

Response: We agree with the reviewer. This limitation is already acknowledged in the Limitations section: "while Benjamini-Hochberg correction was applied for differential expression, other exploratory analyses (e.g., drug sensitivity) were not corrected for multiple testing, increasing type I error risk." We have therefore not made additional changes, as we believe this statement adequately addresses the concern.

Reproducibility. The datasets are publicly available, which is appreciated. However, reproducibility would be substantially improved if the authors also made the analysis scripts or code publicly available through an appropriate repository.

Response: We agree with the reviewer. All analysis scripts have been uploaded to a public GitHub repository and are now available at: <https://github.com/zhengwl1/code_melanoma>

The link has been added to the Data Availability section of the manuscript (Lines 624-625).

Minor comments

Please define all scoring approaches more clearly, including the rationale for selecting the genes used to calculate the CAF, immune, proliferation, and desmosome scores.

Response: We agree with the reviewer. All module scores were calculated using a standardized approach: for each cell or ROI, we first extracted the log-normalized expression values of the genes in each predefined gene set, then computed the mean expression across all genes in the set. The gene sets were defined based on the following rationales: (1) CAF score genes (COL1A2, DCN, COL6A2, COL1A1, FBLN1) were selected from fibroblast-specific extracellular matrix genes that showed high and specific expression in fibroblasts in our scRNA-seq data (Fig. 1B) and are established CAF markers in the literature; (2) immune score genes (PTPRC, CD68, CD4, CD8A, CSF1R, CD74) were chosen to represent major immune cell populations; (3) proliferation score genes (MKI67, PCNA, TOP2A, CDK1, CCNB1, AURKA, BIRC5) were selected based on their established roles in cell cycle progression; and (4) desmosome score genes (DSC1, DSC2, DSC3, DSG1, DSG2, DSG3, DSP, PKP1, PKP2, PKP3, JUP, PERP) were derived from the gene set used in Baron et al. (manuscript reference 19) to represent the desmosome complex. All scoring details are now more clearly described in the Methods section (sections 2.6, 2.7, and 2.8) (Lines 155-160; Line 167; Line 171; Line 179).

The manuscript would benefit from additional details regarding parameter selection for clustering and module score analyses to facilitate reproducibility.

Response: We agree with the reviewer. We have expanded the Methods section to provide detailed parameter selection for clustering and module score analyses. Specifically:

1. For scRNA-seq clustering: PCs were selected based on the elbow plot of variance explained; resolution was set to 0.5 to separate major cell types; UMAP was performed with n.neighbors = 30 and min.dist = 0.3 (section 2.3) (Lines 127-131).
2. For spatial clustering: resolution was set to 0.5 to yield biologically interpretable spatial clusters; dimensionality reduction was based on the first 10 PCs; Louvain clustering was performed with default parameters (section 2.5) (Lines 148-151).
3. For melanoma subclustering: resolution was set to 0.3 to avoid over-splitting the small number of melanoma cells (n = 588); UMAP was performed with n.neighbors = 20 and min.dist = 0.3; dimensionality reduction was based on the first 10 PCs (section 2.10) (Lines 195-201).
4. For module score analyses: scores were calculated using the Seurat AddModuleScore function, and the threshold of 0.1 was chosen to minimize false-positive cell type assignments (section 2.4) (Lines 138-141).

Several sections of the Discussion could be shortened, as some interpretations extend beyond the presented evidence.

Response: We agree with the reviewer. We have shortened several sections of the Discussion to improve conciseness. Specifically, we condensed the comparison with pan-cancer CAF studies and removed the "First, Second, Third" enumeration in the clinical implications paragraph (Lines 522-527; Lines 540-548). We have also ensured that all interpretations remain within the scope of the presented evidence.

Minor grammatical and stylistic edits would further improve readability, although the manuscript is generally written in clear English.

Response: We thank the reviewer for the positive comment on the clarity of our manuscript. We have carefully proofread the entire text and made minor grammatical and stylistic corrections to improve readability. All changes are highlighted in the revised manuscript.

Overall, this is a potentially valuable study that provides an integrated view of the melanoma tumor microenvironment using publicly available transcriptomic datasets. Addressing the points above would strengthen the manuscript and improve the robustness of the conclusions.

Response: We thank the reviewer for the positive assessment of our study and for the constructive suggestions, which have helped us strengthen the manuscript.

Reviewer #2: Dear Editor,
I have reviewed the manuscript PONE-D-26-26256, entitled "Spatial compartmentalization of melanoma cell states reveals CAF-associated tumor cells with enhanced proliferative capacity," by Dr. Zheng and colleagues.
This study presents an interesting integration of single-cell and spatial transcriptomics to investigate the role of Cancer-Associated Fibroblasts (CAFs) in the melanoma tumor microenvironment. The topic is timely and relevant, and the integration of spatial transcriptomics with single-cell data represents a valuable approach. The manuscript is generally well organized, and the analyses are clearly presented.
While the study has merit, I have concerns that should be addressed before the manuscript is suitable for publication.

Major comments
1. The CAF score is overly simplistic and may not adequately represent CAF heterogeneity. Why did the authors choose a simple five-gene CAF signature instead of validated CAF subtype signatures? How were this specific gene set chosen? Were other well-established CAF markers (e.g., FAP, PDPN, α-SMA) or published CAF signatures considered? A justification for this specific selection is needed to ensure the score comprehensively captures CAF heterogeneity.
Could the observed associations be specific to matrix CAFs rather than CAFs as a whole? Have the authors considered validating their findings using published CAF subtype signatures?

Response: We thank the reviewer for this important comment. We have revised the manuscript to clarify the rationale for our five-gene CAF score. These genes (COL1A2, DCN, COL6A2, COL1A1, FBLN1) were selected because they were among the most highly expressed and consistently detected fibroblast markers across all three scRNA-seq samples and represent core ECM-related genes commonly associated with CAF activation. We acknowledge that this score captures a pan-CAF signature rather than subtype-specific heterogeneity; however, we validated the CAF grouping using alternative CAF markers (FAP, PDPN, THY1, POSTN), confirming the robustness of our stratification.

We also agree that our score is most closely aligned with the mCAF subtype, as it is based on ECM-related genes. We have added a statement in the Discussion acknowledging that the observed associations may be specific to mCAFs rather than CAFs as a whole, and that future studies using subtype-specific signatures may provide additional resolution.

All changes are highlighted in the revised manuscript (Methods section 2.6, Lines 154-160 and Discussion paragraph 5, Lines 535-538).

2. The GeoMx Digital Spatial Profiling data are used for spatial analysis. As noted in the limitations, a ROI can contain a mixed population of cells. Could the negative correlation between CAF score and immune score simply reflect the segregation of stromal and immune regions, rather than a functional CAF-mediated exclusion mechanism? Can the authors provide spatial tissue images or UMAP projections for individual samples to visually demonstrate the spatial segregation of CAF-high and immune-high regions?

Response: We thank the reviewer for raising this important point. We agree that the observed negative correlation between CAF score and immune score could reflect spatial compartmentalization—the segregation of CAF-rich and immune-rich regions due to tissue architecture—rather than active CAF-mediated exclusion. We have added a statement in the Discussion acknowledging this alternative interpretation, while noting that the enrichment of ECM-related genes in CAF-high regions and the spatial co-expression of FN1-ITGB1 are consistent with a potential functional role for CAFs. We also clarify that our correlative data cannot distinguish between these possibilities, and that functional experiments are needed to establish causality.

Regarding spatial visualization, Figure 3B already provides UMAP projections showing the spatial distribution of immune scores stratified by CAF status across all ROIs, visually demonstrating the segregation of CAF-high (low immune scores) and CAF-low (high immune scores) regions.

All changes are highlighted in the revised manuscript (Discussion paragraph 2; Lines 457-464).

3. The observed spatial co-expression of FN1 and ITGB1 is intriguing. Considering that FN1 is predominantly produced by mCAFs and not iCAFs, do the authors believe that their pan-CAF score diluted this signal? Would an mCAF-specific analysis not provide a much stronger validation of the ECM-receptor signaling mechanism they propose for immune exclusion? Are there other ECM-receptor pairs (e.g., COL1A1-ITGA1, COL1A2-ITGA2) explored? If so, did any show stronger correlations than FN1-ITGB1
Response: We thank the reviewer for this insightful comment. We agree that our pan-CAF score may have diluted the FN1 signal, as FN1 is predominantly produced by mCAFs. To address this point, we examined additional ECM-receptor pairs, including COL1A1-ITGA1 and COL1A2-ITGA2, using the same spatial transcriptomic data. Interestingly, COL1A1-ITGA1 showed a stronger correlation (ρ = 0.192, p = 7.4 × 10⁻⁹) than FN1-ITGB1 (ρ = 0.100, p = 0.003), while COL1A2-ITGA2 showed no significant positive correlation (ρ = -0.081, p = 0.016). These results suggest that collagen-integrin interactions, particularly involving COL1A1, may also contribute to the ECM-immune signaling axis, and support the reviewer's suggestion that mCAF-specific analyses could yield stronger signals. We have added these findings to the Results section (3.3) and revised the Discussion accordingly (Lines 464-476). FN1-ITGB1 remained significantly correlated, supporting its relevance as an ECM-receptor pair in our dataset. We also acknowledge that future studies using mCAF-specific signatures may provide additional resolution.

4. The authors identify six melanoma cell states via unsupervised clustering but do not explain how this number was determined. Could you elaborate on the biological and/or marker-based rationale for the number of states (k=6)? Was a specific method (e.g., the Elbow method) used to determine the optimal number of clusters?
Response: We thank the reviewer for this question. We have added clarification to the Methods section regarding the determination of cluster number. The resolution parameter (0.3) was selected by testing values from 0.1 to 0.8 and evaluating the resulting clusters for biological interpretability based on known melanoma cell state markers (e.g., pigmentation, proliferation, Wnt signaling). A lower resolution was chosen to avoid over-splitting the limited number of melanoma cells (n = 588). Resolution = 0.3 yielded six clusters with distinct and biologically meaningful marker gene expression profiles (Figure 4A-B), supporting the relevance of this clustering solution. All changes are highlighted in the revised manuscript (Methods section 2.10) (Lines 195-201).

5. The authors reported that State 4 (CAF-associated) has significantly higher proliferative capacity than State 5 (immune-associated). This is an important finding. However, the "proliferation score" is based on three genes (MKI67, MCM4, PCNA). Is it possible that State 4 and State 5 are simply at different stages of the cell cycle rather than intrinsically different in proliferative capacity?
Response: We thank the reviewer for this important point. We agree that the observed difference could theoretically reflect cell cycle phase rather than intrinsic proliferative capacity. To address this concern, we performed a sensitivity analysis using an independent set of proliferation-associated genes (AURKA, BIRC5, CDK2, CCNA2, CDC20), as shown in Supplementary Figure 4C. The proliferation scores derived from this alternative gene set were strongly correlated with our original three-gene score, and the difference between State 4 and State 5 remained significant. This consistency across two independent gene sets supports the robustness of our finding and suggests that State 4 cells are indeed more proliferative, rather than simply being at a different cell cycle phase.

6. A key finding is that the CAF score is negatively correlated with the immune score in spatial data (Fig. 3A) but positively correlated in the TCGA bulk data (Fig. 5A). The manuscript attributes this to the "averaging effect" of bulk RNA-seq, which is a plausible explanation. Could you provide a more detailed exploration of this discordance?
Response: We thank the reviewer for this suggestion. We have expanded our discussion of the discordance between spatial and bulk correlations. As detailed in Discussion paragraph 4, we now clarify that bulk RNA-seq averages signals across heterogeneous tissue compartments, potentially masking the spatial segregation observed at single-ROI resolution. We also note that the positive correlation at the bulk level may reflect global tumor inflammation rather than local CAF-immune interactions, and that these complementary perspectives are not contradictory but rather highlight that CAF-immune relationships operate at different spatial scales. All changes are highlighted in the revised manuscript (Lines 509-516).

7. The drug sensitivity analysis, where a higher CAF score correlates with cisplatin sensitivity and trametinib resistance, is compelling. Could you provide more details on how this could be clinically applied? For example, in the GDSC and TCGA datasets, did you look at any known mutational status (e.g., BRAF V600E) that could be a confounder in this association?
The CAF score aggregates all fibroblasts into a single metric, masking the well-documented functional heterogeneity of CAF subtypes (e.g., iCAFs, mCAFs). Given that mCAFs are classically associated with drug resistance through ECM-mediated integrin signaling, while iCAFs can modulate inflammation, do the authors believe that using a pan-CAF score is appropriate for drug sensitivity analyses? Could the observed cisplatin sensitivity and trametinib resistance be attributed to opposite CAF subtypes, and if so, would the correlations not be substantially strengthened by using validated subtype-specific signatures (e.g., a specific mCAF signature)?
Response: We thank the reviewer for these important points. We acknowledge that we did not examine BRAF V600E or other mutation status in the GDSC or TCGA datasets. The drug sensitivity analysis was intended as an exploratory screening to identify potential associations between the CAF signature and drug response, not as a definitive predictor of clinical outcome. We have added this as a limitation (Discussion paragraph 4 [Lines 516-520] and Limitations [Lines 568-570]).

We also agree that our pan-CAF score may obscure subtype-specific contributions to drug sensitivity. The observed correlations with cisplatin and trametinib may be driven by distinct CAF subtypes (e.g., mCAF vs iCAF). We acknowledge this limitation and now explicitly state that future studies using validated subtype-specific signatures may provide stronger and more mechanistically informative correlations (Discussion paragraphs 5 [Lines 535-538] and 6 [Lines 548-551]).

8. Based on your CAF score, which marker or combination of markers would you suggest for immunohistochemical (IHC) validation of this signature in routine pathology samples? This would be a significant step towards clinical translation.
Response: We thank the reviewer for this forward-looking suggestion. For immunohistochemical validation of the CAF signature in routine pathology samples, we propose using a combination of COL1A2 and DCN. COL1A2 showed the highest differential expression between CAF-high and CAF-low regions (log2FC = 1.34) in our spatial data, and DCN is a well-established fibroblast marker with widely available commercial antibodies. Combined staining of these two markers could provide a practical approach for identifying CAF-rich regions in melanoma tissue sections. We have added this suggestion to the Discussion (Lines 551-557).

Minor comments
1. Please explain why only three scRNA-seq melanoma samples were analyzed while 892 ROIs were available for spatial analysis.
Response: We thank the reviewer for this question. The scRNA-seq data were derived from three primary melanoma samples based on the availability of matched single-cell data in the original study [19]. The spatial transcriptomics dataset included 892 ROIs from eight samples, providing broader spatial coverage. We have added this clarification to the Methods section (2.2) (Lines 104-105).

2. Please justify the choice of module score cutoff (0.1) for cell annotation.
Response: We thank the reviewer for this question. As now stated in the Methods section (2.4) (Lines 138-141), the module score threshold of 0.1 was chosen empirically to minimize false-positive cell type assignments while retaining confident annotation of major cell populations. Module scores were calculated using the Seurat AddModuleScore function, which computes the average expression of each gene set minus the average expression of a control set.

3. Why was the Louvain resolution set to 0.3 for melanoma subclustering? Was cluster stability evaluated?

Response: We thank the reviewer for this question. As stated in the Methods section (2.10) (Lines 195-201), the resolution parameter was selected by testing values from 0.1 to 0.8. Resolution = 0.3 was chosen because it yielded clusters with distinct and biologically interpretable marker gene expression profiles (Figure 4A-B) without over-splitting the limited number of melanoma cells (n = 588). Cluster stability was assessed based on the consistency and biological interpretability of marker gene expression across the resulting clusters.

4. Were batch effects between melanoma samples corrected before integration?

Response: We thank the reviewer for this question. Batch effects between the three melanoma samples were corrected using the Harmony algorithm prior to downstream analysis. We assessed sample mixing before and after correction; consistent with the high inter-sample correlation (>0.77; Supplementary Figure 1E), Harmony correction did not substantially alter mixing (paired t-test, p = 0.56), indicating that batch effects were minimal in our dataset. We have added this information to the Methods section (2.3) (Line 131).

5. Were fibroblast markers removed before calculating differential expressions to avoid circularity between CAF score and CAF-associated genes?

Response: We thank the reviewer for this methodological concern. The five genes used for CAF score calculation were not excluded from the differential expression analysis, as the goal was to characterize the full transcriptional landscape of CAF-high versus CAF-low regions, rather than to identify novel CAF-specific markers. We acknowledge that these genes were included among the significantly upregulated genes, but they represented only a small fraction of the total (5 out of 192), indicating that the observed transcriptional differences were not driven solely by the CAF score definition. We have added this clarification to the Methods section (2.9) (Lines 188-190).

6. Some figures report linear regression lines despite Spearman correlation analysis. Please explain this choice.

Response: We thank the reviewer for this question. The linear regression lines in Figures 3A, 3G, and 5A are included for visualization purposes to illustrate the overall trend in the data, while statistical significance was assessed using Spearman correlation (non-parametric). We have clarified this in the corresponding figure legends.

7. At line 303, the citation to Figure 1A appears to be incorrect. It should refer to Figure 4A.

Response: We thank the reviewer for catching this error. We have corrected the citation from Figure 1A to Figure 4A in the Results section (3.4), where we describe the subclustering of melanoma cells.
