# Supplementary material for: Spatial compartmentalization of melanoma cell states reveals CAF-associated tumor cells with enhanced proliferative capacity
Source: PLoS One. 2026 Sep 15;21(9):e0358235. doi: 10.1371/journal.pone.0358235 (PMC13577406; doi:10.1371/journal.pone.0358235)
Supplement: Peer review history

# Peer review: Decision Letter 0

**DOI:** 10.1371/journal.pone.0358235.r001

**Authors:** Xing-Xiong An

22 Jul 2026

Dear Dr. Zheng,

Thank you for submitting your manuscript to PLOS ONE. After careful consideration, we feel that it has merit but does not fully meet PLOS ONE’s publication criteria as it currently stands. Therefore, we invite you to submit a revised version of the manuscript that addresses the points raised during the review process.

Please submit your revised manuscript by Sep 20 2026 11:59PM. If you will need more time than this to complete your revisions, please reply to this message or contact the journal office at plosone@plos.org. When you're ready to submit your revision, log on to https://www.editorialmanager.com/pone/ and select the 'Submissions Needing Revision' folder to locate your manuscript file.

- A letter that responds to each point raised by the academic editor and reviewer(s). You should upload this letter as a separate file labeled 'Response to Reviewers'.
- A marked-up copy of your manuscript that highlights changes made to the original version. You should upload this as a separate file labeled 'Revised Manuscript with Track Changes'.
- An unmarked version of your revised paper without tracked changes. You should upload this as a separate file labeled 'Manuscript'.

If you would like to make changes to your financial disclosure, please include your updated statement in your cover letter. Guidelines for resubmitting your figure files are available below the reviewer comments at the end of this letter.

If applicable, we recommend that you deposit your laboratory protocols in protocols.io to enhance the reproducibility of your results. Protocols.io assigns your protocol its own identifier (DOI) so that it can be cited independently in the future. For instructions see: https://journals.plos.org/plosone/s/submission-guidelines#loc-laboratory-protocols. Additionally, PLOS ONE offers an option for publishing peer-reviewed Lab Protocol articles, which describe protocols hosted on protocols.io. Read more information on sharing protocols at https://plos.org/protocols?utm_medium=editorial-email&utm_source=authorletters&utm_campaign=protocols.

As the corresponding author, your ORCID iD is verified in the submission system and will appear in the published article. PLOS supports the use of ORCID, and we encourage all coauthors to register for an ORCID iD and use it as well. Please encourage your coauthors to verify their ORCID iD within the submission system before final acceptance, as unverified ORCID iDs will not appear in the published article. *Only*  the individual author can complete the verification step; PLOS staff *cannot*  verify ORCID iDs on behalf of authors.

We look forward to receiving your revised manuscript.

Kind regards,

Xing-Xiong An, M.D.

Academic Editor

PLOS One

Journal Requirements:

When submitting your revision, we need you to address these additional requirements.

1. Please ensure that your manuscript meets PLOS ONE's style requirements, including those for file naming. The PLOS ONE style templates can be found at

https://journals.plos.org/plosone/s/file?id=wjVg/PLOSOne_formatting_sample_main_body.pdf and https://journals.plos.org/plosone/s/file?id=ba62/PLOSOne_formatting_sample_title_authors_affiliations.pdf

2. Please note that PLOS One has specific guidelines on code sharing for submissions in which author-generated code underpins the findings in the manuscript. In these cases, we expect all author-generated code to be made available without restrictions upon publication of the work. Please review our guidelines at https://journals.plos.org/plosone/s/materials-and-software-sharing#loc-sharing-code and ensure that your code is shared in a way that follows best practice and facilitates reproducibility and reuse.

3. Your ethics statement should only appear in the Methods section of your manuscript. If your ethics statement is written in any section besides the Methods, please move it to the Methods section and delete it from any other section. Please ensure that your ethics statement is included in your manuscript, as the ethics statement entered into the online submission form will not be published alongside your manuscript.

4. Please include captions for your Supporting Information files at the end of your manuscript, and update any in-text citations to match accordingly. Please see our Supporting Information guidelines for more information: http://journals.plos.org/plosone/s/supporting-information.

5. We are unable to open your Supporting Information file [code.rar]. Please kindly revise as necessary and re-upload.

6. If the reviewer comments include a recommendation to cite specific previously published works, please review and evaluate these publications to determine whether they are relevant and should be cited. There is no requirement to cite these works unless the editor has indicated otherwise.

[Note: HTML markup is below. Please do not edit.]

Reviewers' comments:

Reviewer's Responses to Questions

**Comments to the Author**

1. Is the manuscript technically sound, and do the data support the conclusions?

Reviewer #1: Partly

Reviewer #2: Yes

**********

2. Has the statistical analysis been performed appropriately and rigorously?

Reviewer #1: Yes

Reviewer #2: Yes

**********

3. Have the authors made all data underlying the findings in their manuscript fully available?

The PLOS Data policy

Reviewer #1: Yes

Reviewer #2: Yes

**********

4. Is the manuscript presented in an intelligible fashion and written in standard English?

Reviewer #1: Yes

Reviewer #2: Yes

**********

Reviewer #1: This manuscript presents an integrated analysis of single-cell RNA sequencing, spatial transcriptomics, TCGA, and drug sensitivity datasets to investigate the role of cancer-associated fibroblasts (CAFs) in the melanoma tumor microenvironment. The study addresses an important topic, and the integration of multiple publicly available datasets is a strength. The manuscript is generally well organized, and the figures are clear and informative. However, several issues should be addressed before the manuscript can be considered for publication.

Major comments

The conclusions are stronger than the evidence presented. The study is entirely computational and observational. While the analyses identify associations between CAF-rich regions, immune exclusion, tumor cell states, and drug sensitivity, they do not establish causality. Statements suggesting that CAFs "create" immune exclusion or that the FN1-ITGB1 axis mediates these effects should be consistently presented as hypotheses or potential mechanisms unless supported by functional validation.

Limited validation. Although TCGA data are used for validation, this represents bulk RNA-seq data and cannot validate the spatial observations directly. Moreover, the positive correlation between CAF score and immune score in TCGA contrasts with the negative correlation observed in the spatial dataset. While the authors discuss this discrepancy, additional discussion is needed to explain how this affects the biological interpretation and the robustness of the proposed model.

Drug sensitivity analysis requires greater caution. Drug sensitivity is inferred from correlations between CAF scores and GDSC melanoma cell line data. Because CAFs are absent from conventional cell lines, it is difficult to interpret CAF-derived signatures as predictors of drug response in this context. The limitations of this analysis should be discussed more explicitly, and conclusions regarding clinical utility should be moderated.

Multiple testing. Although the manuscript states that Benjamini-Hochberg correction was applied where indicated, several exploratory analyses, including the drug sensitivity analysis, appear to rely on unadjusted p-values. The potential for false-positive findings should be discussed, and multiple-testing correction should be considered where appropriate.

Reproducibility. The datasets are publicly available, which is appreciated. However, reproducibility would be substantially improved if the authors also made the analysis scripts or code publicly available through an appropriate repository.

Minor comments

Please define all scoring approaches more clearly, including the rationale for selecting the genes used to calculate the CAF, immune, proliferation, and desmosome scores.

The manuscript would benefit from additional details regarding parameter selection for clustering and module score analyses to facilitate reproducibility.

Several sections of the Discussion could be shortened, as some interpretations extend beyond the presented evidence.

Minor grammatical and stylistic edits would further improve readability, although the manuscript is generally written in clear English.

Overall, this is a potentially valuable study that provides an integrated view of the melanoma tumor microenvironment using publicly available transcriptomic datasets. Addressing the points above would strengthen the manuscript and improve the robustness of the conclusions.

Reviewer #2: Dear Editor,

I have reviewed the manuscript PONE-D-26-26256, entitled "Spatial compartmentalization of melanoma cell states reveals CAF-associated tumor cells with enhanced proliferative capacity," by Dr. Zheng and colleagues.

This study presents an interesting integration of single-cell and spatial transcriptomics to investigate the role of Cancer-Associated Fibroblasts (CAFs) in the melanoma tumor microenvironment. The topic is timely and relevant, and the integration of spatial transcriptomics with single-cell data represents a valuable approach. The manuscript is generally well organized, and the analyses are clearly presented.

While the study has merit, I have concerns that should be addressed before the manuscript is suitable for publication.

Major comments

1. The CAF score is overly simplistic and may not adequately represent CAF heterogeneity. Why did the authors choose a simple five-gene CAF signature instead of validated CAF subtype signatures? How were this specific gene set chosen? Were other well-established CAF markers (e.g., FAP, PDPN, α-SMA) or published CAF signatures considered? A justification for this specific selection is needed to ensure the score comprehensively captures CAF heterogeneity.

Could the observed associations be specific to matrix CAFs rather than CAFs as a whole? Have the authors considered validating their findings using published CAF subtype signatures?

2. The GeoMx Digital Spatial Profiling data are used for spatial analysis. As noted in the limitations, a ROI can contain a mixed population of cells. Could the negative correlation between CAF score and immune score simply reflect the segregation of stromal and immune regions, rather than a functional CAF-mediated exclusion mechanism? Can the authors provide spatial tissue images or UMAP projections for individual samples to visually demonstrate the spatial segregation of CAF-high and immune-high regions?

3. The observed spatial co-expression of FN1 and ITGB1 is intriguing. Considering that FN1 is predominantly produced by mCAFs and not iCAFs, do the authors believe that their pan-CAF score diluted this signal? Would an mCAF-specific analysis not provide a much stronger validation of the ECM-receptor signaling mechanism they propose for immune exclusion? Are there other ECM-receptor pairs (e.g., COL1A1-ITGA1, COL1A2-ITGA2) explored? If so, did any show stronger correlations than FN1-ITGB1

4. The authors identify six melanoma cell states via unsupervised clustering but do not explain how this number was determined. Could you elaborate on the biological and/or marker-based rationale for the number of states (k=6)? Was a specific method (e.g., the Elbow method) used to determine the optimal number of clusters?

5. The authors reported that State 4 (CAF-associated) has significantly higher proliferative capacity than State 5 (immune-associated). This is an important finding. However, the "proliferation score" is based on three genes (MKI67, MCM4, PCNA). Is it possible that State 4 and State 5 are simply at different stages of the cell cycle rather than intrinsically different in proliferative capacity?

6. A key finding is that the CAF score is negatively correlated with the immune score in spatial data (Fig. 3A) but positively correlated in the TCGA bulk data (Fig. 5A). The manuscript attributes this to the "averaging effect" of bulk RNA-seq, which is a plausible explanation. Could you provide a more detailed exploration of this discordance?

7. The drug sensitivity analysis, where a higher CAF score correlates with cisplatin sensitivity and trametinib resistance, is compelling. Could you provide more details on how this could be clinically applied? For example, in the GDSC and TCGA datasets, did you look at any known mutational status (e.g., BRAF V600E) that could be a confounder in this association?

The CAF score aggregates all fibroblasts into a single metric, masking the well-documented functional heterogeneity of CAF subtypes (e.g., iCAFs, mCAFs). Given that mCAFs are classically associated with drug resistance through ECM-mediated integrin signaling, while iCAFs can modulate inflammation, do the authors believe that using a pan-CAF score is appropriate for drug sensitivity analyses? Could the observed cisplatin sensitivity and trametinib resistance be attributed to opposite CAF subtypes, and if so, would the correlations not be substantially strengthened by using validated subtype-specific signatures (e.g., a specific mCAF signature)?

8. Based on your CAF score, which marker or combination of markers would you suggest for immunohistochemical (IHC) validation of this signature in routine pathology samples? This would be a significant step towards clinical translation.

Minor comments

1. Please explain why only three scRNA-seq melanoma samples were analyzed while 892 ROIs were available for spatial analysis.

2. Please justify the choice of module score cutoff (0.1) for cell annotation.

3. Why was the Louvain resolution set to 0.3 for melanoma subclustering? Was cluster stability evaluated?

4. Were batch effects between melanoma samples corrected before integration?

5. Were fibroblast markers removed before calculating differential expressions to avoid circularity between CAF score and CAF-associated genes?

6. Some figures report linear regression lines despite Spearman correlation analysis. Please explain this choice.

7. At line 303, the citation to Figure 1A appears to be incorrect. It should refer to Figure 4A.

**********

what does this mean?). If published, this will include your full peer review and any attached files.

If you choose “no”, your identity will remain anonymous but your review may still be made public.

**Do you want your identity to be public for this peer review?**  For information about this choice, including consent withdrawal, please see our Privacy Policy

Reviewer #1: No

Reviewer #2: **Yes:** Bruna Da Silva Soley

**********

[NOTE: If reviewer comments were submitted as an attachment file, they will be attached to this email and accessible via the submission site. Please log into your account, locate the manuscript record, and check for the action link "View Attachments". If this link does not appear, there are no attachment files.]

To ensure your figures meet our technical requirements, please review our figure guidelines: https://journals.plos.org/plosone/s/figures

You may also use PLOS’s free figure tool, NAAS, to help you prepare publication quality figures: https://journals.plos.org/plosone/s/figures#loc-tools-for-figure-preparation.

NAAS will assess whether your figures meet our technical requirements by comparing each figure against our figure specifications.

# Author comment: Author response to Decision Letter 1

**DOI:** 10.1371/journal.pone.0358235.r002

28 Jul 2026

Dear Dr. An and Reviewer,

We thank the editor and reviewers for their thoughtful comments and constructive suggestions on our manuscript. We have carefully considered all the points raised and have revised the manuscript accordingly. Below we provide point-by-point responses to each comment. All changes in the revised manuscript are highlighted in yellow

We believe the revised manuscript is substantially strengthened and hope it now meets the standards for publication in PLOS ONE.

Sincerely,

Weilong Zheng, MD

Corresponding author

Reviewer #1: This manuscript presents an integrated analysis of single-cell RNA sequencing, spatial transcriptomics, TCGA, and drug sensitivity datasets to investigate the role of cancer-associated fibroblasts (CAFs) in the melanoma tumor microenvironment. The study addresses an important topic, and the integration of multiple publicly available datasets is a strength. The manuscript is generally well organized, and the figures are clear and informative. However, several issues should be addressed before the manuscript can be considered for publication.

Major comments

The conclusions are stronger than the evidence presented. The study is entirely computational and observational. While the analyses identify associations between CAF-rich regions, immune exclusion, tumor cell states, and drug sensitivity, they do not establish causality. Statements suggesting that CAFs "create" immune exclusion or that the FN1-ITGB1 axis mediates these effects should be consistently presented as hypotheses or potential mechanisms unless supported by functional validation.

Response: We agree with the reviewer's concern. We have revised the language throughout the manuscript to ensure that all conclusions are presented as hypotheses or potential mechanisms rather than causal claims. Specifically, we have:

1. Changed "demonstrating" to "suggesting" (Discussion, first paragraph, Line 432)

2. Changed "uncovered" to "observed" (Discussion, first paragraph, Line 438)

3. Changed "provide direct evidence" to "show" (Discussion, second paragraph, Line 444)

4. Changed "demonstrate" to "show" (Conclusion, Line 573)

5. Changed "demonstrate that CAFs create" to "suggest that CAFs may contribute to" (Results 3.3) (Line 331)

Limited validation. Although TCGA data are used for validation, this represents bulk RNA-seq data and cannot validate the spatial observations directly. Moreover, the positive correlation between CAF score and immune score in TCGA contrasts with the negative correlation observed in the spatial dataset. While the authors discuss this discrepancy, additional discussion is needed to explain how this affects the biological interpretation and the robustness of the proposed model.

Response: We agree with the reviewer. We have revised our description of TCGA analysis from "validation" to "complementary analysis" throughout the manuscript (Line 11; Lines 378-379; Line 440).

We have also expanded our discussion of the discordance between spatial and bulk findings:" This discordance does not undermine the spatial observations but rather highlights that bulk RNA-seq averages signals across tumor and stromal compartments, potentially cancelling out spatially opposed signals. Importantly, the positive correlation at the bulk level may reflect global tumor inflammation rather than local CAF-immune interactions. Collectively, these findings suggest that spatial resolution is essential for dissecting CAF-immune relationships, and that bulk-level correlations should not be interpreted as contradicting spatial observations (Lines 509-516)."

Drug sensitivity analysis requires greater caution. Drug sensitivity is inferred from correlations between CAF scores and GDSC melanoma cell line data. Because CAFs are absent from conventional cell lines, it is difficult to interpret CAF-derived signatures as predictors of drug response in this context. The limitations of this analysis should be discussed more explicitly, and conclusions regarding clinical utility should be moderated.

Response: We agree with the reviewer. We have moderated the language throughout the drug sensitivity analysis and added explicit acknowledgment of the limitation that GDSC cell lines lack CAFs. Specifically, we have:

1. Revised the Abstract conclusion to reflect that these findings warrant further investigation in CAF-containing systems (Lines 26-27).

2. Revised Results 3.6 to present these findings as hypothesis-generating (Lines 423-427).

3. Added a statement in the Discussion acknowledging that cell line-based correlations may not reflect the drug response of CAF-rich tumors (Lines 509-516).

4. Added a dedicated limitation in the Limitations section stating that drug sensitivity predictions require validation in CAF-containing experimental systems (Lines 560-564).

Multiple testing. Although the manuscript states that Benjamini-Hochberg correction was applied where indicated, several exploratory analyses, including the drug sensitivity analysis, appear to rely on unadjusted p-values. The potential for false-positive findings should be discussed, and multiple-testing correction should be considered where appropriate.

Response: We agree with the reviewer. This limitation is already acknowledged in the Limitations section: "while Benjamini-Hochberg correction was applied for differential expression, other exploratory analyses (e.g., drug sensitivity) were not corrected for multiple testing, increasing type I error risk." We have therefore not made additional changes, as we believe this statement adequately addresses the concern.

Reproducibility. The datasets are publicly available, which is appreciated. However, reproducibility would be substantially improved if the authors also made the analysis scripts or code publicly available through an appropriate repository.

Response: We agree with the reviewer. All analysis scripts have been uploaded to a public GitHub repository and are now available at: https://github.com/zhengwl1/code_melanoma

The link has been added to the Data Availability section of the manuscript (Lines 624-625).

Minor comments

Please define all scoring approaches more clearly, including the rationale for selecting the genes used to calculate the CAF, immune, proliferation, and desmosome scores.

Response: We agree with the reviewer. All module scores were calculated using a standardized approach: for each cell or ROI, we first extracted the log-normalized expression values of the genes in each predefined gene set, then computed the mean expression across all genes in the set. The gene sets were defined based on the following rationales: (1) CAF score genes (COL1A2, DCN, COL6A2, COL1A1, FBLN1) were selected from fibroblast-specific extracellular matrix genes that showed high and specific expression in fibroblasts in our scRNA-seq data (Fig. 1B) and are established CAF markers in the literature; (2) immune score genes (PTPRC, CD68, CD4, CD8A, CSF1R, CD74) were chosen to represent major immune cell populations; (3) proliferation score genes (MKI67, PCNA, TOP2A, CDK1, CCNB1, AURKA, BIRC5) were selected based on their established roles in cell cycle progression; and (4) desmosome score genes (DSC1, DSC2, DSC3, DSG1, DSG2, DSG3, DSP, PKP1, PKP2, PKP3, JUP, PERP) were derived from the gene set used in Baron et al. (manuscript reference 19) to represent the desmosome complex. All scoring details are now more clearly described in the Methods section (sections 2.6, 2.7, and 2.8) (Lines 155-160; Line 167; Line 171; Line 179).

The manuscript would benefit from additional details regarding parameter selection for clustering and module score analyses to facilitate reproducibility.

Response: We agree with the reviewer. We have expanded the Methods section to provide detailed parameter selection for clustering and module score analyses. Specifically:

1. For scRNA-seq clustering: PCs were selected based on the elbow plot of variance explained; resolution was set to 0.5 to separate major cell types; UMAP was performed with n.neighbors = 30 and min.dist = 0.3 (section 2.3) (Lines 127-131).

2. For spatial clustering: resolution was set to 0.5 to yield biologically interpretable spatial clusters; dimensionality reduction was based on the first 10 PCs; Louvain clustering was performed with default parameters (section 2.5) (Lines 148-151).

3. For melanoma subclustering: resolution was set to 0.3 to avoid over-splitting the small number of melanoma cells (n = 588); UMAP was performed with n.neighbors = 20 and min.dist = 0.3; dimensionality reduction was based on the first 10 PCs (section 2.10) (Lines 195-201).

4. For module score analyses: scores were calculated using the Seurat AddModuleScore function, and the threshold of 0.1 was chosen to minimize false-positive cell type assignments (section 2.4) (Lines 138-141).

Several sections of the Discussion could be shortened, as some interpretations extend beyond the presented evidence.

Response: We agree with the reviewer. We have shortened several sections of the Discussion to improve conciseness. Specifically, we condensed the comparison with pan-cancer CAF studies and removed the "First, Second, Third" enumeration in the clinical implications paragraph (Lines 522-527; Lines 540-548). We have also ensured that all interpretations remain within the scope of the presented evidence.

Minor grammatical and stylistic edits would further improve readability, although the manuscript is generally written in clear English.

Response: We thank the reviewer for the positive comment on the clarity of our manuscript. We have carefully proofread the entire text and made minor grammatical and stylistic corrections to improve readability. All changes are highlighted in the revised manuscript.

Overall, this is a potentially valuable study that provides an integrated view of the melanoma tumor microenvironment using publicly available transcriptomic datasets. Addressing the points above would strengthen the manuscript and improve the robustness of the conclusions.

Response: We thank the reviewer for the positive assessment of our study and for the constructive suggestions, which have helped us strengthen the manuscript.

Reviewer #2: Dear Editor,

I have reviewed the manuscript PONE-D-26-26256, entitled "Spatial compartmentalization of melanoma cell states reveals CAF-associated tumor cells with enhanced proliferative capacity," by Dr. Zheng and colleagues.

This study presents an interesting integration of single-cell and spatial transcriptomics to investigate the role of Cancer-Associated Fibroblasts (CAFs) in the melanoma tumor microenvironment. The topic is timely and relevant, and the integration of spatial transcriptomics with single-cell data represents a valuable approach. The manuscript is generally well organized, and the analyses are clearly presented.

While the study has merit, I have concerns that should be addressed before the manuscript is suitable for publication.

Major comments

1. The CAF score is overly simplistic and may not adequately represent CAF heterogeneity. Why did the authors choose a simple five-gene CAF signature instead of validated CAF subtype signatures? How were this specific gene set chosen? Were other well-established CAF markers (e.g., FAP, PDPN, α-SMA) or published CAF signatures considered? A justification for this specific selection is needed to ensure the score comprehensively captures CAF heterogeneity.

Could the observed associations be specific to matrix CAFs rather than CAFs as a whole? Have the authors considered validating their findings using published CAF subtype signatures?

Response: We thank the reviewer for this important comment. We have revised the manuscript to clarify the rationale for our five-gene CAF score. These genes (COL1A2, DCN, COL6A2, COL1A1, FBLN1) were selected because they were among the most highly expressed and consistently detected fibroblast markers across all three scRNA-seq samples and represent core ECM-related genes commonly associated with CAF activation. We acknowledge that this score captures a pan-CAF signature rather than subtype-specific heterogeneity; however, we validated the CAF grouping using alternative CAF markers (FAP, PDPN, THY1, POSTN), confirming the robustness of our stratification.

We also agree that our score is most closely aligned with the mCAF subtype, as it is based on ECM-related genes. We have added a statement in the Discussion acknowledging that the observed associations may be specific to mCAFs rather than CAFs as a whole, and that future studies using subtype-specific signatures may provide additional resolution.

All changes are highlighted in the revised manuscript (Methods section 2.6, Lines 154-160 and Discussion paragraph 5, Lines 535-538).

2. The GeoMx Digital Spatial Profiling data are used for spatial analysis. As noted in the limitations, a ROI can contain a mixed population of cells. Could the negative correlation between CAF score and immune score simply reflect the segregation of stromal and immune regions, rather than a functional CAF-mediated exclusion mechanism? Can the authors provide spatial tissue images or UMAP projections for individual samples to visually demonstrate the spatial segregation of CAF-high and immune-high regions?

Response: We thank the reviewer for raising this important point. We agree that the observed negative correlation between CAF score and immune score could reflect spatial compartmentalization—the segregation of CAF-rich and immune-rich regions due to tissue architecture—rather than active CAF-mediated exclusion. We have added a statement in the Discussion acknowledging this alternative interpretation, while noting that the enrichment of ECM-related genes in CAF-high regions and the spatial co-expression of FN1-ITGB1 are consistent with a potential functional role for CAFs. We also clarify that our correlative data cannot distinguish between these possibilities, and that functional experiments are needed to establish causality.

Regarding spatial visualization, Figure 3B already provides UMAP projections showing the spatial distribution of immune scores stratified by CAF status across all ROIs, visually demonstrating the segregation of CAF-high (low immune scores) and CAF-low (high immune scores) regions.

All changes are highlighted in the revised manuscript (Discussion paragraph 2; Lines 457-464).

3. The observed spatial co-expression of FN1 and ITGB1 is intriguing. Considering that FN1 is predominantly produced by mCAFs and not iCAFs, do the authors believe that their pan-CAF score diluted this signal? Would an mCAF-specific analysis not provide a much stronger validation of the ECM-receptor signaling mechanism they propose for immune exclusion? Are there other ECM-receptor pairs (e.g., COL1A1-ITGA1, COL1A2-ITGA2) explored? If so, did any show stronger correlations than FN1-ITGB1

Response: We thank the reviewer for this insightful comment. We agree that our pan-CAF score may have diluted the FN1 signal, as FN1 is predominantly produced by mCAFs. To address this point, we examined additional ECM-receptor pairs, including COL1A1-ITGA1 and COL1A2-ITGA2, using the same spatial transcriptomic data. Interestingly, COL1A1-ITGA1 showed a stronger correlation (ρ = 0.192, p = 7.4 × 10⁻⁹) than FN1-ITGB1 (ρ = 0.100, p = 0.003), while COL1A2-ITGA2 showed no significant positive correlation (ρ = -0.081, p = 0.016). These results suggest that collagen-integrin interactions, particularly involving COL1A1, may also contribute to the ECM-immune signaling axis, and support the reviewer's suggestion that mCAF-specific analyses could yield stronger signals. We have added these findings to the Results section (3.3) and revised the Discussion accordingly (Lines 464-476). FN1-ITGB1 remained significantly correlated, supporting its relevance as an ECM-receptor pair in our dataset. We also acknowledge that future studies using mCAF-specific signatures may provide additional resolution.

4. The authors identify six melanoma cell states via unsupervised clustering but do not explain how this number was determined. Could you elaborate on the biological and/or marker-based rationale for the number of states (k=6)? Was a specific method (e.g., th

Attachment Submitted filename: Response letter.docx

# Peer review: Decision Letter 1

**DOI:** 10.1371/journal.pone.0358235.r003

**Authors:** Xing-Xiong An

30 Aug 2026

Spatial compartmentalization of melanoma cell states reveals CAF-associated tumor cells with enhanced proliferative capacity

PONE-D-26-26256R1

Dear Dr. Zheng,

We’re pleased to inform you that your manuscript has been judged scientifically suitable for publication and will be formally accepted for publication once it meets all outstanding technical requirements.

Within one week, you’ll receive an e-mail detailing the required amendments. When these have been addressed, you’ll receive a formal acceptance letter and your manuscript will be scheduled for publication.

An invoice will be generated when your article is formally accepted. Please note, if your institution has a publishing partnership with PLOS and your article meets the relevant criteria, all or part of your publication costs will be covered. Please make sure your user information is up-to-date by logging into Editorial Manager at Editorial Manager® and clicking the ‘Update My Information' link at the top of the page. For questions related to billing, please contact billing support.

If your institution or institutions have a press office, please notify them about your upcoming paper to help maximize its impact. If they’ll be preparing press materials, please inform our press team as soon as possible -- no later than 48 hours after receiving the formal acceptance. Your manuscript will remain under strict press embargo until 2 pm Eastern Time on the date of publication. For more information, please contact onepress@plos.org.

Kind regards,

Xing-Xiong An, M.D.

Academic Editor

PLOS One

Additional Editor Comments (optional):

Reviewers' comments:

Reviewer's Responses to Questions

**Comments to the Author**

Reviewer #1: All comments have been addressed

Reviewer #2: All comments have been addressed

**********

2. Is the manuscript technically sound, and do the data support the conclusions?

Reviewer #1: Yes

Reviewer #2: Yes

**********

3. Has the statistical analysis been performed appropriately and rigorously?

Reviewer #1: Yes

Reviewer #2: Yes

**********

4. Have the authors made all data underlying the findings in their manuscript fully available?

The PLOS Data policy

Reviewer #1: Yes

Reviewer #2: Yes

**********

5. Is the manuscript presented in an intelligible fashion and written in standard English?

Reviewer #1: Yes

Reviewer #2: Yes

**********

Reviewer #1: I thank the authors for their thorough and thoughtful responses to the reviewers' comments. The revised manuscript has been substantially improved. The authors have appropriately moderated causal interpretations, expanded the discussion of the differences between spatial and bulk transcriptomic analyses, clarified the methodology, improved the reproducibility of the study by making the analysis code publicly available, and added important limitations regarding the interpretation of the drug sensitivity analyses.

The additional methodological details and expanded discussion strengthen the manuscript, and I appreciate the authors' careful attention to the concerns raised during the initial review. The conclusions are now appropriately aligned with the observational nature of the analyses.

I have no further major comments and believe that the manuscript is suitable for publication in its current form.

Reviewer #2: (No Response)

**********

what does this mean?). If published, this will include your full peer review and any attached files.

If you choose “no”, your identity will remain anonymous but your review may still be made public.

**Do you want your identity to be public for this peer review?**  For information about this choice, including consent withdrawal, please see our Privacy Policy

Reviewer #1: No

Reviewer #2: No

**********

# Editor report: Acceptance letter

**DOI:** 10.1371/journal.pone.0358235.r004

**Authors:** Xing-Xiong An

PONE-D-26-26256R1

PLOS One

Dear Dr. Zheng,

I'm pleased to inform you that your manuscript has been deemed suitable for publication in PLOS One. Congratulations! Your manuscript is now being handed over to our production team.

At this stage, our production department will prepare your paper for publication. This includes ensuring the following:

* All references, tables, and figures are properly cited

* All relevant supporting information is included in the manuscript submission,

* There are no issues that prevent the paper from being properly typeset

You will receive further instructions from the production team, including instructions on how to review your proof when it is ready. Please keep in mind that we are working through a large volume of accepted articles, so please give us a few days to review your paper and let you know the next and final steps.

Lastly, if your institution or institutions have a press office, please let them know about your upcoming paper now to help maximize its impact. If they'll be preparing press materials, please inform our press team within the next 48 hours. Your manuscript will remain under strict press embargo until 2 pm Eastern Time on the date of publication. For more information, please contact onepress@plos.org.

You will receive an invoice from PLOS for your publication fee after your manuscript has reached the completed accept phase. If you receive an email requesting payment before acceptance or for any other service, this may be a phishing scheme. Learn how to identify phishing emails and protect your accounts at https://explore.plos.org/phishing.

If we can help with anything else, please email us at customercare@plos.org.

Thank you for submitting your work to PLOS One and supporting open access.

Kind regards,

PLOS One Editorial Office Staff

on behalf of

Dr. Xing-Xiong An

Academic Editor

PLOS One
